# Systematic review and meta-analysis on the utility of Interferon-gamma release assays for the diagnosis of *Mycobacterium tuberculosis* infection in children: a 2013 update

**DOI:** 10.1186/1471-2334-14-S1-S6

**Published:** 2014-01-08

**Authors:** S Sollai, L Galli, M de Martino, E Chiappini

**Affiliations:** 1 Department of Health Sciences, Meyer Children University Hospital, University of Florence, Florence, Italy

## Abstract

**Background:**

Previous meta-analyses regarding the performance of interferon-gamma release assays (IGRAs) for tuberculosis diagnosis in children yielded contrasting results, probably due to different inclusion/exclusion criteria.

**Methods:**

We systematically searched PubMed, EMBASE and Cochrane databases and calculated pooled estimates of sensitivities and specificities of QuantiFERON-TB Gold In Tube (QFT-G-IT), T-SPOT.TB, and tuberculin skin test (TST). Several sub-analysis were performed: stratification by background (low income *vs.* high income countries); including only microbiological confirmed TB cases; including only studies performing a simultaneous three-way comparison of the three tests, and including immunocompromised children.

**Results:**

Overall, 31 studies (6183 children) for QFT-G-IT, 14 studies (2518 children) for T-SPOT.TB and 34 studies (6439 children) for TST were included in the analyses. In high income countries QFT-G-IT sensitivity was 0.79 (95%IC: 0.75-0.82) considering all the studies, 0.78 (95%CI:0.70-0.84) including only studies performing a simultaneous three-way comparison and 0.86 (95%IC 0.81-0.90) considering only microbiologically confirmed studies. In the same analyses T-SPOT.TB sensitivity was 0.67 (95%IC 0.62-0.73); 0.76 (95%CI: 0.68 to 0.83); and 0.79 (95%IC 0.69-0.87), respectively. In low income countries QFT-G-IT pooled sensitivity was significantly lower: 0.57 (95%IC:0.52-0.61), considering all the studies, and 0.66 (95%IC 0.55-0.76) considering only microbiologically confirmed cases; while T-SPOT.TB sensitivity was 0.61 (95%IC 0.57-0.65) overall, but reached 0.80 (95%IC 0.73-0.86) in microbiologically confirmed cases. In microbiologically confirmed cases TST sensitivity was similar: 0.86 (95%IC 0.79-0.91) in high income countries, and 0.74 (95%IC 0.68-0.80) in low income countries. Higher IGRAs specificity with respect to TST was observed in high income countries (97-98% *vs.* 92%) but not in low income countries (85-93% *vs.* 90%).

**Conclusions:**

Both IGRAs showed no better performance than TST in low income countries.

## Background

Diagnosis of paediatric tuberculosis infection remains a challenging issue. Tuberculin skin test (TST) has several limitations: sensitivity may be influenced by the child’s age and immunologic status, bacille Calmette-Guérin (BCG) vaccination or non-tuberculosis mycobacterium-infections; in case of repeated tests a booster effect can occur and a double access to a health care facility is needed. Nevertheless, infiltrate measurement may be operator-dependent [1-4]. Interferon gamma release assays (IGRAs), including the commercially available assays QuantiFERON®-TB Gold (QFT-G; Cellestis, Australia), QuantiFERON®-TB Gold In-Tube (QTF-G-IT, Cellestis, Australia), and the T-SPOT TB (T-SPOT®, Oxford Immunotec Ltd. UK), have been extensively used for the diagnosis of tuberculosis infection in adults.

IGRAs allow detection of circulating T-cells responsive to specific *Mycobacterium tuberculosis* antigens, which are absent in BCG and many non-tuberculosis mycobacteria, and displayed similar sensitivity and higher specificity than TST in adults (table 1) [5-12]. However, IGRA performance in paediatric populations is still under debate and caution is recommended for their use and interpretation in children [13]. Some authors reported a sub-optimal IGRA sensitivity in children [14], but this finding has not been confirmed by others [8]. Five meta-analyses have previously assessed IGRA sensitivity and specificity in children but reported pooled estimates largely differ (Table 1) [1,5,9-11]. Such discordances may be due to different inclusion/exclusion criteria and, thus, different characteristics of the study populations (i.e. proportion of immunocompromised/HIV infected and/or young children included). The setting is also relevant. IGRA performance is influenced by the child’s immunologic status, which, in turn, may be impaired by several conditions commonly observed in low income countries, such as malnutrition, HIV-infection, and elminthiasis [1-3]. Hereby, we report results of an updated meta-analysis regarding IGRA performance in children, focusing on settings, age, and immunologic status of the study children.

**Table 1:** Sensitivity and specificity of Interferon-gamma release assays (IGRAs) and tuberculin skin test (TST) in the previous published meta-analyses [1,5-12]

	TST	QFN-G-IT	T-SPOT.TB	Population	Number of studies considered	Author, year of publication
**Sensitivity**	0.70 (95%CI 0.67-0.72)	0.81 (95%CI 0.78-0.89)	0.84 (95%CI 0.81-0.87)	Adults	124	*Diel, 2010*
	
	0.77 (95%CI 0.71-0.82)	0.78 (95% CI 73-82)	0.90 (95%CI 0.86-0.93)	Adults	20	*Pai, 2008*
	
		0.80 (95%CI 0.75-0.91)	0.81 (95CI 0.78-0.84)	Adults	27	*Sester, 2010*
	
	0.80 (95%CI 0.70-0.90)	0.83(95%CI 0.75-0.92)	0.84(95%CI 0.63-1.00)	Children	32	*Mandalakas, 2011*
	
	0.71(95%CI 0.67-0.75)	0.70 (95%CI 0.65-0.75)	0.62 (95%CI 0.57-0.67)	Children	16	*Sun, 2011*
	
	0.82(95%CI 0.72-0.93)	0.79 (95%CI 0.70-0.89)	0.74 (95%CI 0.59-0.90)	Children	11	*Chiappini, 2012*
	
		0.77 (95%CI 0.64-0.73)		Adults and Children	11	*Dheda, 2009*
	
		0.66 (95%IC 0.53-0.78)		Children	6	*Machingaidze, 2011*
	
	0.71 (95%CI 0.65-0.74)	0.76 (95%CI 0.70-0.83)	0.88 (95%CI 0.81-0.95)	Adults and Children	58	*Menzies, 2007*

**Specificity**		0.99 (95%CI 0.98-1.00)	0.86 (95%CI 0.81-0.90)	Adults	124	*Diel, 2010*
	
	0.59 (95%CI 0.46-0.73)	0.96 (95%CI 94-98)	0.93 (95%CI 0.86-1.00)	Adults	20	*Pai, 2008*
	
		0.82 (95%CI 0.70-0.91)	0.82 (95%CI 0.78-0.86)	Adults	27	*Sester, 2010*
	
	0.85 (95%CI 0.69-1.00)	0.91 (95% CI 0.78-1.00)	0.94 (95%CI 0.87-1.00)	Children	32	*Mandalakas, 2011*
	
	0.56 (95% CI 0.50-0.61)	1.00 (95%CI 0.84-1.00)	0.90 (95%CI 0.86-0.93)	Children	16	*Sun, 2011*
	
	0.83 (95%CI 0.74-0.92)	0.95 (95%CI 0.93-0.97)	0.96 (95%CI 0.95-1.00)	Children	8	*Chiappini, 2012*
	
	0.66 (95%CI 0.46-0.86)	0.97 (95%CI 0.96-0.99)	0.92 (95%CI 0.86-0.99)	Adults and Children	58	*Menzies, 2007*

## Methods

A literature search using multiple keywords and standardized terminology in Medline, EMBASE and Cochrane databases dating back to their inception up to and through June 7th, 2013, as summarized in Additional file 1, appendix 1. Inclusion and exclusion criteria, extraction of data and assessment of study quality are reported in Additional file 1. In particular only studies evaluating QuantiFERON®-TB Gold In-Tube (QTF-G-IT, Cellestis, Australia), and/or T-SPOT TB (T-SPOT®, Oxford Immunotec Ltd. UK) in comparison to TST were included while studies using QFT-G were excluded.

## Statistical methods

For each included study, we computed and calculated sensitivity or specificity (and 95% CIs) and summarized the results in forest plots. Random-effects meta-analysis was performed using MetaDiSc®, Meta-analysis of Diagnostic and Screening tests, Version 1.4 [15]. Studies were weighted by total sample size to pool estimates of sensitivity and specificity across the studies. Chi-square test was used to evaluate the presence of statistically significant heterogeneity across studies, whose variance proportion attributable to between studies heterogeneity was expressed calculating the I^2^ statistic.

## Results

For the analysis of sensitivity, 31 studies (20 conducted in high income countries and 11 in low income countries) for QFT-G-IT, including 6183 children [3,16-44], 14 studies (9 conducted in high income countries and 5 conducted in low income countries for T-SPOT.TB including 2518 children [10,14,16,18,19,22,23,37,39,45-48,50] and 34 studies (18 studies conducted in high income countries and 16 studies conducted in low income countries) for TST including 6439 children [14,17-35,37-43,45-47,49,50] were included.

**Figure 1:**
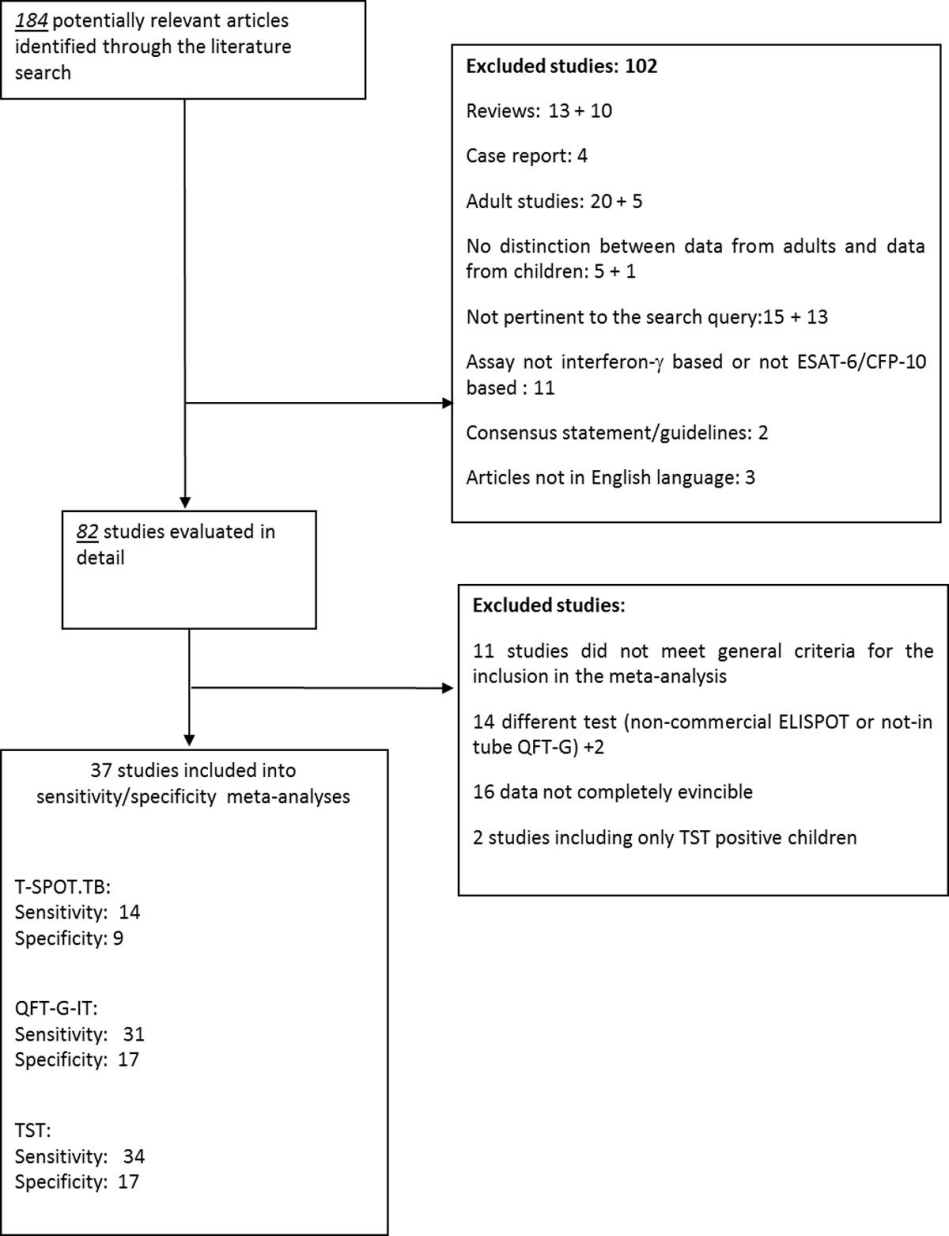
**Selection of studies for the meta-analysis.** *Inclusion/exclusion criteria as reported in the text and in additional file 1.

The specificity analysis included 17 studies, overall including 3844 children (11 conducted in high income countries and 6 in low income countries) using QFT-G-IT [16-22,24-27,30,32,34,37,43], 9 studies overall including 1296 children (6 conducted in high income countries and 3 in low income countries) using T-SPOT.TB [16,18,19,22,23,45,46,48,49], and 17 studies overall including 3548 children (10 conducted in high income countries and 7 conducted in low income countries) using TST [17-23,25-27,30,32,34,37,45-47], as summarized in Figure 1.

## Pooled IGRAs and TST sensitivity and specificity in high and low income countries

**Figure 2:**
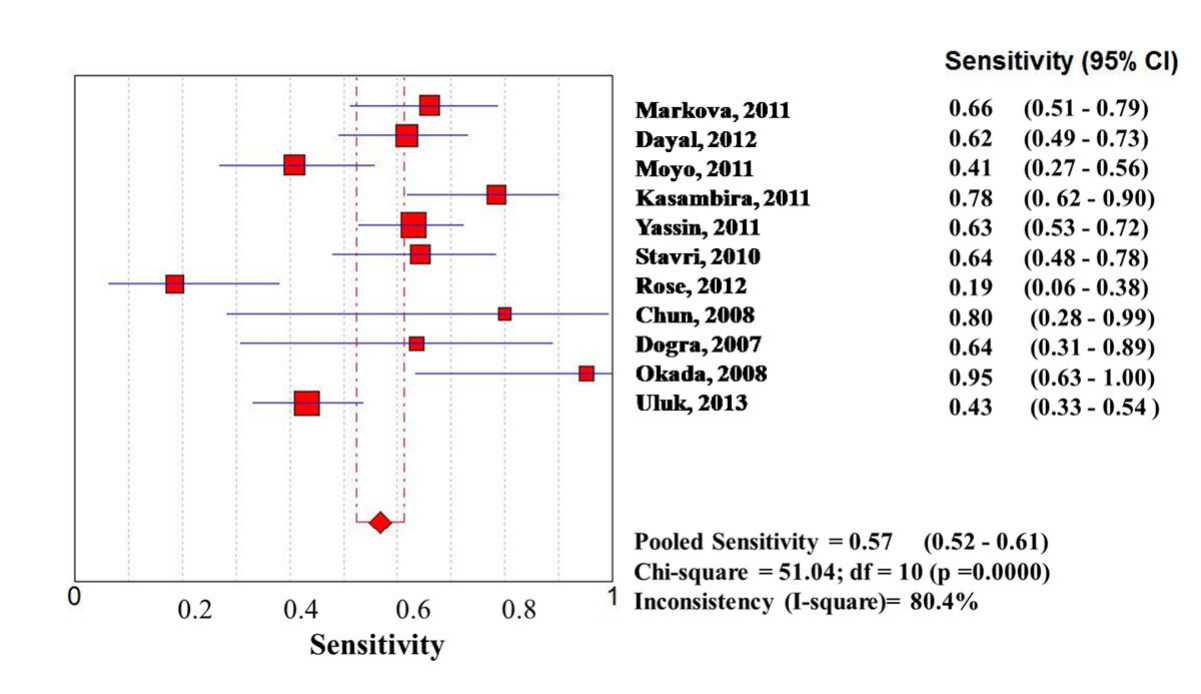
**Forest plot of sensitivity of QuantiFERON-TB Gold In Tube (QFT-G-IT) in low-income countries.** The squares are single study estimates and the error bars represent the 95% confidence intervals (95% CIs). The diamonds are pooled estimates.

**Figure 3:**
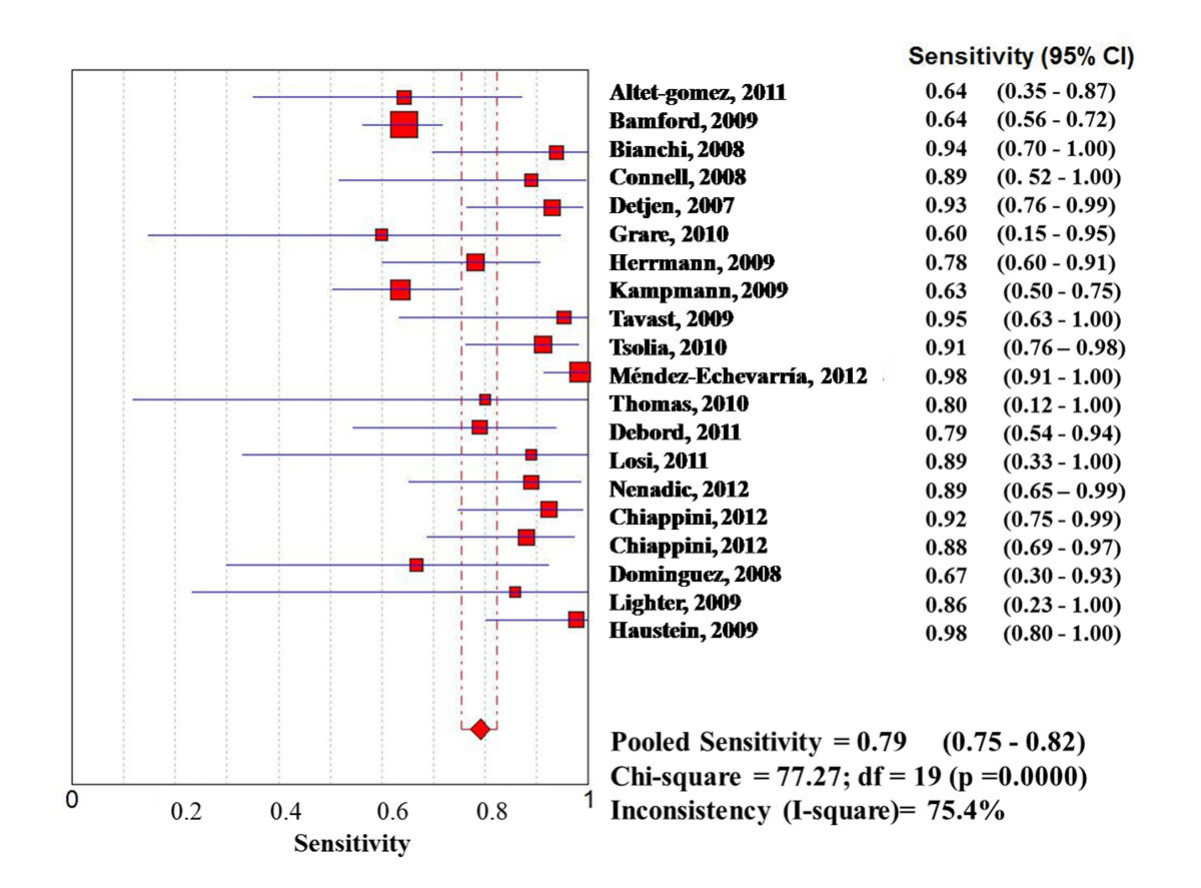
**Forest plot of sensitivity of QuantiFERON-TB Gold In Tube (QFT-G-IT) in high-income countries.** The squares are single study estimates and the error bars represent the 95% confidence intervals (95% CIs). The diamonds are pooled estimates.

**Figure 4:**
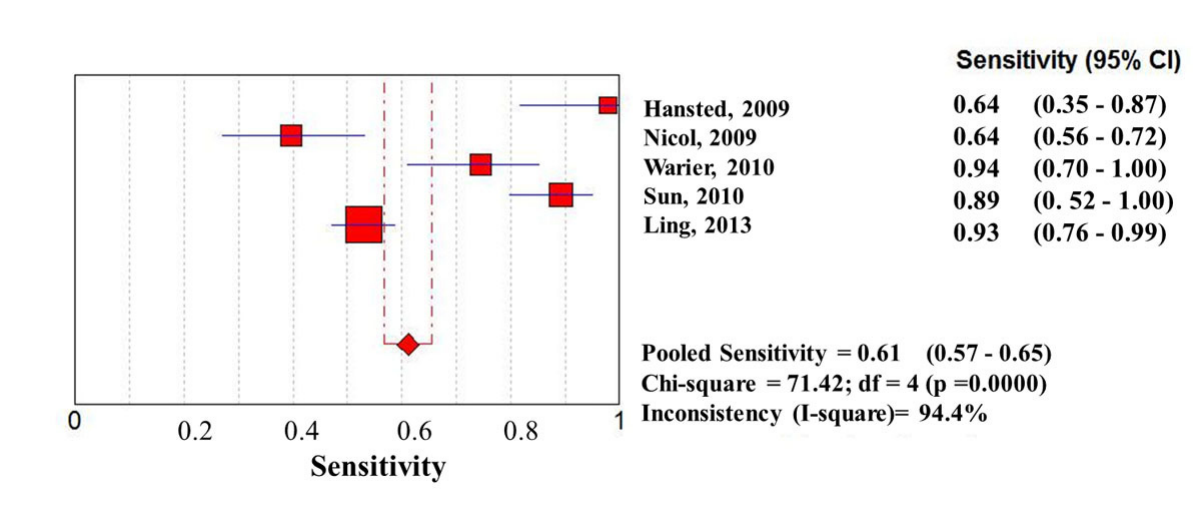
**Forest plot of sensitivity of T-SPOT.TB in low-income countries.** The squares are single study estimates and the error bars represent the 95% confidence intervals (95% CIs). The diamonds are pooled estimates.

**Figure 5:**
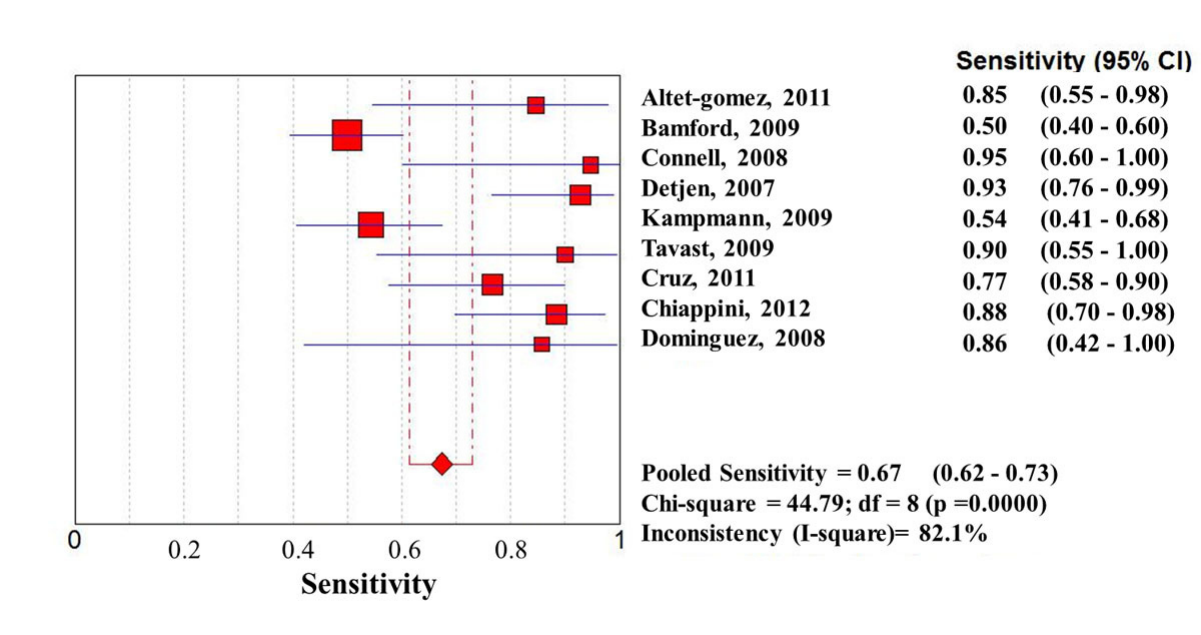
**Forest plot of sensitivity of T-SPOT.TB in high-income countries.** The squares are single study estimates and the error bars represent the 95% confidence intervals (95% CIs). The diamonds are pooled estimates.

**Figure 6:**
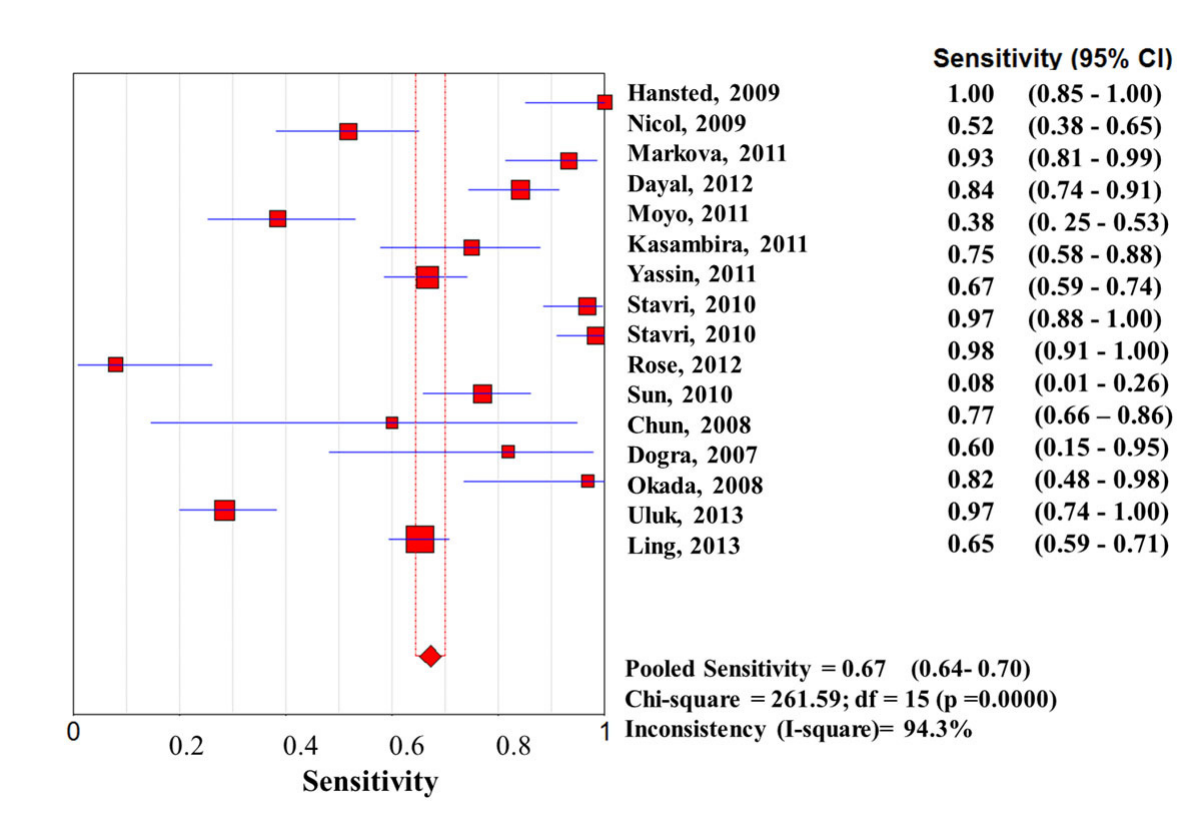
**Forest plot of sensitivity of tuberculin skin test (TST) in low-income countries.** The squares are single study estimates and the error bars represent the 95% confidence intervals (95% CIs). The diamonds are pooled estimates.

Considering the whole study population, significantly higher sensitivities of QFT-G-IT and TST in high- than in low-income countries were observed, with no difference between them, while T-SPOT.TB sensitivity was particularly low in both settings (0.67 [95%IC 0.62-0.73] in high income countries, and 0.61 [95%IC 0.57-0.65] in low income countries) (Figures 1, 2, 3, 4, 5, 6).

**Figure 7:**
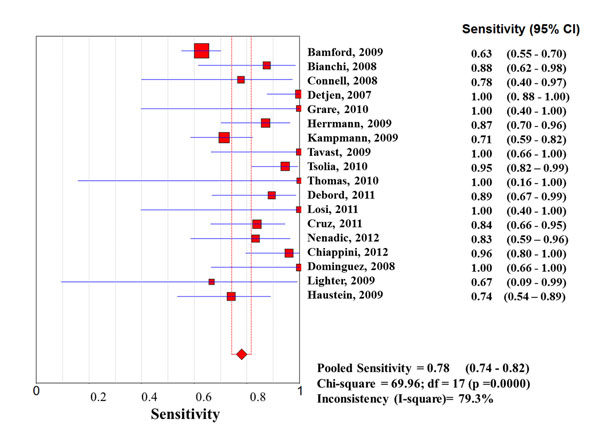
**Forest plot of sensitivity of tuberculin skin test (TST) in high-income countries.** The squares are single study estimates and the error bars represent the 95% confidence intervals (95% CIs). The diamonds are pooled estimates.

In particular, QFT-G-IT sensitivity was 0.79 (95%IC: 0.75-0.82) in high income countries, and 0.57 (95%IC 0.52-0.61) in low income countries, and TST pooled sensitivity was 0.78 (95%IC 0.74-0.82) in high income countries, and 0.67 (95%IC 0.64-0.70) in low income countries.

Specificity of the three test was similar in low income countries while both QFT-G-IT and T-SPOT.TB displayed higher specificity than TST in high income countries. In particular, QFT-G-IT specificity was 0.97 (95%IC 0.96-0.98) in high income countries, and 0.85 (95%IC 0.82-0.88) in low income countries. T-SPOT.TB pooled specificity was 0.98 (95%IC 0.96-0.99) in high income countries, and 0.93 (95%IC 0.87-0.96) in low income countries. TST specificity was 0.92 (95%IC 0.89-0.93) in high income countries, and 0.90 (95%IC 0.87-0.92) in low income countries.

## Sub-analysis for sensitivity including only microbiologically confirmed cases

Data regarding microbiologically confirmed cases were available in 16 studies (11 studies conducted in high income countries and 5 studies conducted in low income countries), overall including 3689 children for QFT-G-T [3,14,17,19,20,22,24,26-29,32,34,37,40,44], 9 studies (4 conducted in high income countries and 5 conducted in low income countries), including 2013 children for T-SPOT.TB [14,19,22,45-50], and 17 studies (10 studies conducted in high income countries and 7 studies conducted in low income countries), including 4494 children for TST [3,14,17,19,20,22,24,26,29,32,37,40,45,47,49,50].

In this analysis sensitivity of the three test was similar (0.81 (95%CI: 0.76-0.85) for QFT-G-IT, 0.80 (95%CI: 0.74-0.84) for T-SPOT.TB, and 0.79 (95%CI: 0.75-0.83) for TST**.**

A sub-analysis in studies conducted in high income and low income countries was performed. In high income countries sensitivity of the three test was confirmed to be similar but IGRAs performance in low income countries was suboptimal. QFT-G-IT pooled sensitivity was 0.86 (95%IC: 0.81-0.90) in high income countries but only 0.66 (95%IC: 0.55-0.76) in low income countries. Even when excluding studies including HIV-infected children, QFT-G-IT pooled sensitivity in low income countries reached only 0.68 (95%IC: 0.55-0.76).

T-SPOT.TB pooled sensitivity was 0.79 (95%IC: 0.69-0.87) in high income countries, and 0.80 (95%IC 0.73-0.86) in low income countries. TST sensitivity was 0.86 (95%IC 0.79-0.91) in high income countries, and 0.74 (95%IC 0.68-0.80) in low income countries (table 2).

**Table 2:** Sensitivities and specificities of Interferon-gamma release assays (IGRAs) and tuberculin skin test (TST) in the present meta-analysis.

		TST	QFN-G-IT	T-SPOT.TB
**Sensitivity**	Low income countries	0.67 (95%CI 0.64-0.70); Chi-square = 261.59; df = 15 (p =0.0000) Inconsistency (I-square)= 94.3%	0.57 (95%CI 0.52-0.61); Chi-square = 51.04; df = 10 (p =0.0000) Inconsistency (I-square)= 80.4%	0.61 (95%CI 0.57-0.65); Chi-square = 71.42; df = 4 (p =0.0000) Inconsistency (I-square)= 94.4%
	
	High income countries	0.78 (95%CI 0.74-0.82); Chi-square = 69.96; df = 17 (p =0.0000) Inconsistency (I-square)= 79.3%	0.79 (95%CI 0.75-0.82); Chi-square **=** 77.27; df = 19 (p =0.0000) Inconsistency (I^2^) = 75.4%	0.67 (95%CI 0.62-0.73); Chi-square = 44.79; df = 8 (p =0.0000) Inconsistency (I-square)= 82.1%
	
	Including only studies using the three assays simoultaneously	0.85 (95%CI 0.78-0.91); Chi-square = 26.01; df = 5 (p =0.0001) Inconsistency (I-square)= 80.8%	0.78 (95%CI 0.70-0.84); Chi-square = 16.85; df = 5 (p =0.0048) Inconsistency (I-square)= 70.3%	0.76 (95%CI 0.68-0.83); Chi-square = 24.94; df = 6 (p =0.0001) Inconsistency (I-square)= 80.0%
	
	Including microbiologically confirmed cases (overall)	0.79 (95%CI 0.75-0.83); Chi-square = 41.56; df = 16 (p =0.0005) Inconsistency (I-square)= 61.5%	0.81 (95%CI 0.76-0.85); Chi-square = 45.51; df = 15 (p =0.0001) Inconsistency (I-square)= 67.0%	0.80 (95%CI 0.73-0.86); Chi-square = 27.31; df = 8 (p =0.0006) Inconsistency (I-square)= 70.7%
	
	Including microbiologically confirmed cases, in low income countries	0.74 (95%CI 0.68-0.80); Chi-square = 20.28; df = 6 (p =0.0025) Inconsistency (I-square)= 70.4%	0.66 (95%CI 0.55-0.76); Chi-square = 6.19; df = 4 (p =0.1857) Inconsistency (I-square)= 35.3% - excluding studies with immunocompromised children: 0.68 (95%IC 0.57-0.79); Chi-square = 3.18; df = 3 (p =0.3644) Inconsistency (I-square)= 5.7%	0.80 (95%CI 0.59-0.90); Chi-square = 17.35; df = 4 (p =0.0017) Inconsistency (I-square)= 76.9%
	
	Including microbiologically confirmed cases, in high income countries	0.86 (0.79-0.91); Chi-square = 14.06; df = 9 (p =0.1203) Inconsistency (I-square)= 36.0%	0.86 (0.81-0.90); Chi-square = 25.78; df = 10 (p =0.0040) Inconsistency (I-square)= 61.2%	0.79 (0.69-0.87); Chi-square = 9.90; df = 3 (p =0.0194) Inconsistency (I-square)= 69.7%
	
	Immunocompromised/HIV infected children	0.54 (95%IC 0.49-0.59); Chi-square = 71.45; df = 3 (p =0.0000) Inconsistency (I-square)= 95.8%	0.47 (95%CI 0.38-0.55); Chi-square = 37.77; df = 2 (p =0.0000) Inconsistency (I-square)= 94.7%	Not evaluable

**Specificity**	Low income countries	0.90 (95%IC 0.87-0.92); Chi-square = 38.57; df = 7 (p =0.0000) Inconsistency (I-square)= 81.9%	0.85 (95%CI 0.82-0.88); Chi-square = 19.15; df = 5 (p =0.0018) Inconsistency (I-square)= 73.9%	0.93 (95%CI 0.87-0.96); Chi-square = 7.75; df = 2 (p =0.0207) Inconsistency (I-square)= 74.2%
	
	High income countries	0.92 (95%CI 0.89-0.93); Chi-square = 125.84; df = 10 (p =0.0000) Inconsistency (I-square)= 92.8%	0.97 (95%CI 0.96-0.98); Chi-square = 38.83; df = 10 (p =0.0000) Inconsistency (I-square)= 74.2%	0.98 (95%CI 0.96-0.99); Chi-square = 12.98; df = 5 (p =0.0235) Inconsistency (I-square)= 61.5%
	
	Including only studies using the three assays simoultaneously (all in high income countries)	0.84 (95%CI 0.79-0.89); Chi-square = 74.74; df = 4 (p =0.0000) Inconsistency (I-square)= 94.6%	0.97 (95% CI 0.93-0.99); Chi-square = 6.05; df = 3 (p =0.1093) Inconsistency (I-square)= 50.4%	0.97 (95%CI 0.93-0.99); Chi-square = 8.47; df = 3 (p =0.0373) Inconsistency (I-square)= 64.6%
	
	Immunocompromised/HIV infected children	0.97 (0.92-0.99); Chi-square = 0.95; df = 1 (p =0.0000) Inconsistency (I-square)= 0.0% Chi-square = 0.95; df = 1 (p =0.0000) Inconsistency (I-square)= 0.0%	0.90 (95%CI 0.81-0.95); Chi-square = 0.00; df = 1 (p =0.9695) Inconsistency (I-square)= 0.0% - only 2 studies could be included -	Not evaluable

## Sub-analysis conducted including only studies performing a simultaneous three-way comparison in the same children (QFT-G-IT; T-SPOT.TB; TST)

Six of the available studies, including 618 children, evaluated all three tests simultaneously in the same children [18,19,22,23,37,39]. All these studies were performed in high income countries. The meta-analytic estimate for sensitivity was 0.78 (95%CI: 0.70-0.84) for QFT-G-IT; 0.76 (95%CI: 0.68-0.83) for T-SPOT.TB, and 0.85 (95%CI: 0.78-0.91) for TST. The meta-analytic estimate for specificity was 0.97 (95%CI: 0.93-0.99) for QFT-G-IT, 0.97 (95%CI: 0.93-0.99) for T-SPOT.TB, and 0.84 (95%CI: 0.79-0.89) for TST (table 2).

## Sub-analysis in immunocompromised/HIV-infected children populations

Overall, 11 studies have assessed utility of IGRAs in paediatric populations including HIV infected children [3,34,43,50,52-58]. However, three of them were excluded because the used tests were the in house-ELISPOT [52,55] and the QFT-G not in-tube assay [58]. In five studies utility of IGRAs in immunocompromised children was evaluated as a screening for LTBI with no case of active TB disease included [53-57], and concordance between tests was evaluated. All the results suggest that due to high rates of discordant and indeterminate results in this population, IGRAs should be interpreted with caution and represent tools of little help for TB infection management for immune-compromised children both in high- and low- prevalence settings [53-57].

Complete data for a specific subgroup-analysis were available in four studies [3,34,43,50]. Haustein and colleagues included in their analysis immunocompromised children with several different pathologic conditions, including malignancies [3]. In three studies utility of QFT-G-IT was compared to TST [3,34,43], while Ling and colleagues compared T-SPOT.TB to TST [50].

The meta-analytic estimate for sensitivity was very low and similar for QFT-G-IT and T.SPOT.TB: 0.47 (95%CI: 0.38-0.55) for QFT-G-IT and 0.54 (95%CI:0.49-0.59) for TST. The meta-analytic estimate for specificity was not performed since only data from 2 studies were available [34,43]. Meta-analytic estimate for sensitivity and specificity for T-SPOT.TB was not performed due to lack of data. In the only study available, Ling and colleagues assessed the incremental value of T-SPOT.TB over and above patient characteristics and conventional tests in 491 smear-negative children from two hospitals in Cape Town, South Africa, founding that cough longer than 2 weeks, fever longer than 2 weeks, night sweats, malaise, history of household contact and HIV status were the most important predictors of culture-confirmed TB and concluding that T-SPOT.TB did not have added value beyond clinical data and conventional tests for diagnosis of TB disease in smear-negative children in a high-burden setting [50].

## Studies with children populations aged ≤ 5 years

Six studies, including 1733 children, included exclusively children aged ≤ 5 years [19,28,30,42,59,60]. In particular, Detjen and colleagues evaluated in 2007 the diagnostic accuracy of TST and 2 IGRAs in a cohort of 73 children (median age: 39 months); comparing 28 children with bacteriologically confirmed TB with children without TB (23 with bacteriologically confirmed non-tuberculous mycobacterial lymphadenitis and 22 with other non-mycobacterial respiratory tract infections) [19]. Specificity of QFT-IT for TB was 1.00 (95%CI: 0.91-1.00), and the specificity of T-SPOT was 0.98 (95% CI: 0.87-1.00). Specificity of TST resulted considerably lower (0.58; 95% CI: 0.42-0.73). The specificity of TST was 0.10 (95% CI: 0.1–0.33) in children with nontuberculous mycobacterial lymphadenitis and 1.00 (95% CI: 0.83–1.00) in children with other non-mycobacterial respiratory tract infections. The sensitivity of both QFT-IT and T-SPOT was 0.93 (95% CI: 0.77–0.99), and the sensitivity of TST was 1.00 (95% CI: 0.88–1.00). Agreement between the two IGRAs was 95.6% (k=0.91). The authors concluded that IGRAs showed high diagnostic value in bacteriologically confirmed childhood TB and when performed in addition to TST they could be able to distinguish -positive TST results caused by non-tuberculous mycobacterial disease [19]. In the same year, Okada and colleagues compared test results of QFT-G-IT and TST in 195 young children household contacts of pulmonary TB patients in Cambodia, founding considerable agreement (k=0.63) between the two tests and that results were not affected by BCG vaccination in a logistic regression analysis [42]. The authors suggested QFT-G-IT may be a substitute for TST in detecting latent TB infection in childhood contacts aged ≤5 years, especially in those who may have a false-positive TST due to BCG vaccination or non-tuberculous mycobacterial infection [42].

Debord and colleagues evaluated QFT-G-IT performance restrospectively in 19 French immunocompetent children (median age: 1.52 years) with active tuberculosis [28]. The rate of indeterminate results was 0/19 and the rates of positivity were 6/10 and 9/9 in <2 and 2- to 5-year-old children, suggesting QFT-G-IT could be a useful tool to improve diagnosis of tuberculosis in association to TST even in young children [29]. In the study conducted by Moyo and colleagues including 397 South African children aged less than 3 years, QFT-G-IT and TST showed notable agreement (k=0.79), however, both tests had low sensitivity for TB disease (38% and 35%) [30]. On contrast, Pavic and colleagues found in 142 Croatian children aged <5 years significant discordance between QFT-G-IT and TST (k=0.59), concluding that both tests should be performed in high-risk children aged <5 years, considering the child infected if either or both tests are positive [59]. Nkurunungi and colleagues found in their cross-sectional study conducted on 907 children screened for LTBI in a high prevalence African setting that T-SPOT.TB results were unstable over a three-week follow-up interval, and that TST compares poorly with T-SPOT.TB, making the categorisation of children as TB-infected or TB-uninfected difficult [60].

Although, in general, results in young children were encouraging, a specific meta-analysis for this subjects, could not be performed as complete data were not available in most studies, except for Detjen *et al*. ‘s study [19].

## Discussion

Data on IGRAs’ performance in children are accumulating. In previous meta-analyses, similarly to data reported in adults, higher IGRA specificity with respect to TST has been reported. However, the reported IGRA sensitivity ranged between 62% and 89% for T-SPOT.TB and 66% and 83% for QFT-G-IT [1,5,9-11]. Differences between IGRA performance in low income and high income countries were evaluated only in one meta-analysis which considered only QFT-G-IT- (and not T-SPOT.TB-) based studies [1]. In that analysis, significantly lower QFT-G-IT sensitivity was observed in high-burden TB settings compared to low-burden TB settings (55% *vs.* 70%). Other authors [10] performed a sub-analysis of paediatric studies by definition of TB cases and reported lower pooled sensitivity including clinical diagnoses TB cases *vs.* microbiologically confirmed TB cases (64% vs. 85% for QFT-G-IT, 66% vs. 76% for T-SPOT.TB and 66% vs. 85% for TST) [10].

Our study is the first paediatric meta-analysis evaluating both QFT-G-IT and T-SPOT.TB performance by setting. Moreover, we were first to present a sub-analysis of studies performing a simultaneous three way comparison using all the tests in the same child, allowing to reduce potential bias due to individual differences.

At a first glance, our meta-analytic results showed a higher sensitivity of QFT-G-IT than TST in high income countries (79% *vs.* 75%), where T-SPOT.TB seems to have lower sensitivity than the two other tests (67%). However this result was not confirmed including only ascertained cases, with a microbiological confirmation. In this sub-analysis T-SPOT.TB sensitivity reached 80% (95%CI: 59-90) while QFT-G-IT sensitivity decreased, but not significantly, to 66% (95%CI:55-76). This finding suggests caution when interpreting results from studies including probable and ascertained TB cases in children, for possible misdiagnoses.

In a further sub-analysis including only studies performing simultaneously the 3 tests, all performed in high income countries, overall including 618 children, no different sensitivity of both IGRAs and TST was observed, while a higher IGRAs specifity was confirmed (97% *vs.* 84%)**.**

Very low sensitivity and specificity were found in the sub-analysis performed with studies in immunocompromised children. The meta-analytic estimate for sensitivity was only 0.54 for TST, and 0.47 for QFT-G-IT, confirming that IGRA results should be still interpreted with caution in immunocompromised children.

Combining all these results, both IGRAs seem to be a reasonable choice in the diagnosis of TB disease in immunocompetent children aged > 5 years in high income countries. In low income-countries and in immunocompromised children IGRAs’ performance is equivalent or inferior to TST. Considerations regarding costs, availability for clinicians and other health workers, patient acceptability, ease of distribution and storage should also be taken into account in this kind of setting. To date, data in the paediatric population aged less than 5 years are limited, and a specific sub-analysis for this category of studies could not be performed as complete data were not evincible from the considered studies [19,28,30,42,59,60].

## Conclusions

In conclusion IGRAs show good promise for improving TB diagnosis only in immunocompetent children aged > 5 years in high income setting. Even in these subjects, however, IGRAs sensitivity was 67-86%, indicating that neither test may rule out nor confirm the certainty of diagnosis and, similarly to the TST, interpretation of results may be difficult. As recently recommended by the NICE guidelines [13], paediatricians while deciding who deserves antitubercular therapy, still have to consider clinical and epidemiological data. Some authors suggest that the combined use of TST and IGRAs might help clinicians by increasing the diagnostic sensitivity to 90%, however interpretation of discordant results is controversial [19].

## Competing interests

All authors declare to have no conflicts of interest.

## Authors' contributions

EC and SS participated in data collection and data analysis. LG and MdM supervised the work. All authors participated in the study design and in writing of the manuscript. EC and SS performed the meta-analysis. All authors approved the final manuscript.

## Supplementary Material

Additional file 1 **Appendix** Contents: Appendix 1: Search strategy. Appendix 2: Studies excluded from the meta-analysis and main exclusion criteria. Appendix 3: Quality assessment of the studies included in the meta-analysis. Appendix 4: Individual-study and pooled estimates for sensitivity, specificity, and summary of the study characteristics. Appendix 5: T-SPOT.TB®, QuantiFERON®-TB Gold In tube and tuberculin skin test data in microbiologically confirmed active tuberculosis cases among the studies included in the sub-analysis to calculate meta-analytic estimates for sensitivity. Appendix 6: Summary of results from relevant studies on Interferon-γ release assays (IGRAs) in children. Click here for file

## Declarations

Funding for this article has come from the Italian Health Ministry /Young Research Project.

This article has been published as part of *BMC Infectious Diseases* Volume 14 Supplement 1, 2014: Highlights in Pediatric Tuberculosis. The full contents of the supplement are available online at http://www.biomedcentral.com/bmcinfectdis/supplements/14/S1
