# Supplementary material for: Systematic review and meta-analysis on the utility of Interferon-gamma release assays for the diagnosis of Mycobacterium tuberculosis infection in children: a 2013 update
Source: BMC Infect Dis. 2014 Jan 8;14(Suppl 1):S6. doi: 10.1186/1471-2334-14-S1-S6 (PMC4016555; doi:10.1186/1471-2334-14-S1-S6)
Supplement: Additional file 1 — Appendix Contents: Appendix 1: Search strategy. Appendix 2: Studies excluded from the meta-analysis and main exclusion criteria. Appendix 3: Quality assessment of the studies included in the meta-analysis. Appendix 4: Individual-study and pooled estimates for sensitivity, specificity, and summary of the study characteristics. Appendix 5: T-SPOT.TB®, QuantiFERON®-TB Gold In tube and tuberculin skin test data in microbiologically confirmed active tuberculosis cases among the studies included in the sub-analysis to calculate meta-analytic estimates for sensitivity. Appendix 6: Summary of results from relevant studies on Interferon-γ release assays (IGRAs) in children. [file 1471-2334-14-S1-S6-S1.doc]

**Appendix 1: Search strategy.**

The search terms used in database searches included: ("child"[MeSH Terms] OR "child"[All Fields] OR "children"[All Fields]) AND ((("interferon-gamma"[MeSH Terms] OR "interferon-gamma"[All Fields] OR ("interferon"[All Fields] AND "gamma"[All Fields]) OR "interferon gamma"[All Fields]) AND release[All Fields] AND ("analysis"[Subheading] OR "analysis"[All Fields] OR "assay"[All Fields] OR "biological assay"[MeSH Terms] OR ("biological"[All Fields] AND "assay"[All Fields]) OR "biological assay"[All Fields])) OR (T-cell-based[All Fields] AND ("analysis"[Subheading] OR "analysis"[All Fields] OR "assay"[All Fields] OR "biological assay"[MeSH Terms] OR ("biological"[All Fields] AND "assay"[All Fields]) OR "biological assay"[All Fields])) OR (antigen-specific[All Fields] AND ("t-lymphocytes"[MeSH Terms] OR "t-lymphocytes"[All Fields] OR "t cell"[All Fields])) OR elispot[All Fields] OR (("enzymes"[MeSH Terms] OR "enzymes"[All Fields] OR "enzyme"[All Fields]) AND Linked[All Fields] AND Immunosorbent[All Fields] AND Spot[All Fields]) OR ((quantiferon[All Fields] OR quantiferonr[All Fields] OR quantiferontb[All Fields] OR quantiferontesten[All Fields]) OR (Quantiferon-TB[All Fields] AND ("gold"[MeSH Terms] OR "gold"[All Fields]))) OR (("interferon-gamma"[MeSH Terms] OR "interferon-gamma"[All Fields] OR ("interferon"[All Fields] AND "gamma"[All Fields]) OR "interferon gamma"[All Fields]) AND release[All Fields] AND ("analysis"[Subheading] OR "analysis"[All Fields] OR "assay"[All Fields] OR "biological assay"[MeSH Terms] OR ("biological"[All Fields] AND "assay"[All Fields]) OR "biological assay"[All Fields]))) AND (("tuberculosis"[MeSH Terms] OR "tuberculosis"[All Fields]) OR ("mycobacterium tuberculosis"[MeSH Terms] OR ("mycobacterium"[All Fields] AND "tuberculosis"[All Fields]) OR "mycobacterium tuberculosis"[All Fields]))

**Appendix 2. Studies excluded from the meta-analysis and main exclusion criteria**

| **Excluded studies** | **Exclusion criteria** |
| --- | --- |
| 1. Dewan PK, Grinsdale J, Liska S *et al. F*easibility, acceptability, a nd cost of tuberculosis testing by whole-blood interferon-gamma assay. BMC Infect Dis 2006;6:47 | **Adult patients** |
| 1. Diel R, Loddenkemper R, Meywald-Walter K *et al.* Comparative performance of tubercoline skin test, QuantiFERON-TB-Gold in Tube assay, and T-Spot. TB test in contact investigations for tuberculosis. Chest 2009;135:1010-1018 | **Adult patients** |
| 1. Diel R, Loddenkemper R, Meywald-Walter K *et al.* Predictive value of a whole blood IFN-gamma assay for the development of active tuberculosis disease after recent infection with Mycobacterium tuberculosis. Am J Respir Crit Care Med 2008;177:1164-1170 | **Adult patients** |
| 1. Dyrhol-Riise AM, Gran G, Wentzel-Larsen T, Blomberg B, et al. Diagnosis and follow-up of treatment of latent tuberculosis; the utility of the QuantiFERON-TB Gold In-tube assay in outpatients from a tuberculosis low-endemic country.MC Infect Dis 2010;10:57. | **Adult patients** |
| 1. Hill PC, Fox A, Jeffries DJ *et al.* Quantitative T cell assay reflects infectious load of Mycobacterium tuberculosis in an endemic case contact model. Clin Infect Dis 2005;40:273-278 | **Adult patients** |
| 1. Hill PC , Brookes RH, Fox A *et al.* Large-scale evaluation of enzyme-linked immunospot assay and skin test for diagnosis of Mycobacterium tuberculosis infection against a gradient of exposure in The Gambia. Clin Infect Dis 2004;38:966-973 | **Adult patients** |
| 1. Jackson-Sillah D, Hill PC *et al.* Screening for tuberculosis among 2381 household contacts of sputum smear-positive cases in The Gambia. Trans R Soc Trop med Hyg 2007;101:594-601 | **Adult patients** |
| 1. Kobashi Y, Mouri K, Miyashita N, et al. QuantiFERON TB-2G test for patients with active tuberculosis stratified by age groups. Scand J Infect Dis 2009;41:841.6 | **Adult patients** |
| 1. Mahomed H, Hughes EJ, Hawkridge T *et al.* Comparison of mantoux skin test with three generations of a whole blood IFN-gamma assay for tuberculosis infection. Int J Tuberc Lung Dis 2006;10:310-316 | **Adult patients** |
| 1. Mantegani P, Piana F, Galli L *et al.* Comparison for commercial and in-house T cell.based assay for the diagnosis of Mycobacterium tuberculosis infection. New Microbiol 2007;30:291-294 | **Adult patients** |
| 1. Mantegani P, Piana F, Codecasa L *et al.* Comparison of an in-house and commercial RD1-based ELISPOT-IFN-gamma assay for the diagnosis of Mycobacterium infection. Clin Med Rers 2006;4:266-272 | **Adult patients** |
| 1. Menzines D, Pai M, Comstock G. Meta-analysis: new test for the diagnosis of latent tuberculosis infection: areas of uncertainty and recommendations for research. Ann Intern Med 2007;146:340-354 | **Adult patients** |
| 1. Nguyen M, Perry S, Parsonnet J. QuantiFERON-TB predicts tubercolin skin test boosting in U.S. foreign-bom. Int J Tuberc Lung Dis 2005;9:985-991 | **Adult patients** |
| 1. Perry S, Sanchez L, Yang S e*t al.* Reproducibility of QuantiFERON-TB gold in-tube assay. Clin Vaccine Immunol 200815:425-432 | **Adult patients** |
| 1. [Soysal A](http://www.ncbi.nlm.nih.gov/pubmed?term=), [Torun T](http://www.ncbi.nlm.nih.gov/pubmed?term=), [Efe S](http://www.ncbi.nlm.nih.gov/pubmed?term=), [Gencer H](http://www.ncbi.nlm.nih.gov/pubmed?term=), [Tahaoglu K](http://www.ncbi.nlm.nih.gov/pubmed?term=), [Bakir M](http://www.ncbi.nlm.nih.gov/pubmed?term=).Evaluation of cut-off values of interferon-gamma-based assays in the diagnosis of M. tuberculosis infection. [Int J Tuberc Lung Dis.](javascript:AL_get(this, 'jour', 'Int J %0D%0ATuberc Lung Dis.');) 2008 ;12:50-56. | **Adult patients** |
| 1. Torres HE, ZApico M, Vivas S *et al.* Clinical performance of antigen-specific interferon-gamma assay for the diagnosis of latent tuberculosis in risk hospital-based populations. Int J Tuberc Ling Dis 2006;10:939-941 | **Adult patients** |
| 1. [Tsiouris SJ](http://www.ncbi.nlm.nih.gov/pubmed?term=), [Coetzee D](http://www.ncbi.nlm.nih.gov/pubmed?term=), [Toro PL](http://www.ncbi.nlm.nih.gov/pubmed?term=), [Austin J](http://www.ncbi.nlm.nih.gov/pubmed?term=), [Stein Z](http://www.ncbi.nlm.nih.gov/pubmed?term=), [El-Sadr W](http://www.ncbi.nlm.nih.gov/pubmed?term=). Sensitivity analysis and potential uses of a novel gamma interferon release assay for diagnosis of tuberculosis. [J Clin Microbiol](javascript:AL_get(this, 'jour', 'J Clin %0D%0AMicrobiol.');) 2006;44:2844-50. | **Adult patients** |
| 1. Yoshiyama T, Harada N, Higuchi K, et al. use of the QuantiFERON-TB-Gold test for screening tuberculosis contacts and predicting active disease. Int J Tuberc Lung Dis 2010;14:819-27 | **Adult patients** |
| 1. Tuuminen T, Sovra S, Lippo K *et al.* feasibility of commercial interferon-gamma-based methods for the diagnosis of latent Mycobacterium tuberculosis infection in Finland, a country of low incidence and high bacilli Calmette-Guèrin vaccination coverage. Clin Microbiol infect 2007;13:836-838 | **Adults patients** |
| 1. Wagstaff Aj, Zellweger JP. T-SPOT TB: an in vitro diagnosis assay measuring T-cell reaction to Mycobacterium tuberculosis-specific antigens. Mol Diagn Ther 2006;10:57-63 | **Adults patients** |
| 1. Kurup SK, Buggage RR, Clarke GL *et al.*Gamma interferon assay as an alternative to PPD skin testing in selected patients with granulomatosis intraocular inflammatory disease. Can J Ophtalmol 2006;41:737-740 | **Case report** |
| 1. Mendez Echevarria A, Vaquero-Artigao F, Gonzalez-Munoz M, et al. Lack of sensitivity of QuantiFERON-TB-gold test in tube in a child with tuberculous meningitis. Pediatr Infect Dis J 2010;29:683-4 | **Case report** |
| 1. Richeldi L. Ewer K, Losi M et al. T-cell-based diagnosis of neonatal multidrug resistant latent tuberculosis infection. Pediatrics 2007;119:e1-e15 | **Case report** |
| 1. Spyridis N, Chakraborty R, Sharland M *et al.* Early diagnosis using an INF-gamma assay in a child with HIV-1 infection and very low CD4 count. Scand J infect Dis 2007;39:919-921 | **Case report** |
| 1. Mack U, Migliori GB, Sester M *et al.* LTBI: latent tuberculosis infection or lasting immune responses to M. tuberculosis? A TBNET consensus statement. Eur Respir J 2009;33:956-973 | **Consensus statement** |
| 1. el Corral H, París SC, Marín et al. Mycobacterium tuberculosis, risk of infection and disease in household contacts of tuberculosis patients in Colombia. PLoS One. 2009;4:e8257. | **Different test** |
| 1. Feske M, Nudelman RJ, Medina M *et al.* Enhancement of human antigen-specific memory T-cell responses by interleukin-7 may improve accuracy in diagnosis tuberculosis. Clin Vaccine Immunol 2008;15:1616-1622 | **Different test** |
| 1. [Fox A](http://www.ncbi.nlm.nih.gov/pubmed?term=), [Jeffries DJ](http://www.ncbi.nlm.nih.gov/pubmed?term=), [Hill PC](http://www.ncbi.nlm.nih.gov/pubmed?term=) *et al.* ESAT-6 and CFP-10 can be combined to reduce the cost of testing for Mycobacterium tuberculosis infection, but CFP-10 responses associate with active disease. Trans R Soc Trop Med Hyg 2007;101:691-698 | **Different test** |
| 1. Gallant CJ, Cobat A, Simkin L, et al. Impact of age and sex on micobacterial immunity in an area of high tuberculosis incidence. Int J Tuberc Lung Dis 2010;14:952-9. | **Different test** |
| 1. Hinks TS, Dosanjh DP, Innes JA et al. Frequencies of region of difference 1 antigen-specific but not purified protein derivative-specific gamma interferon-secreting T cells correlate with the presence of tuberculosis disease but do not distinguish recent from remote latent infections. Infect Immun 2009;77:5486-95. | **Different test** |
| 1. Krummel B, Strassburg A, Emst M, et al. Potential role for IL-2 ELISpot in differentiating recent and remote infection in tuberculosis conctact tracing. Plos One 2010;5:e11670 | **Different test** |
| 1. La Torre I, De Souza-Galvao M, Ruiz-Manzano J, Lacoma A, et al  Evaluating the non-tuberculous mycobacteria effect in the tuberculosis infection diagnosis. Eur Respir J. 2010;35:338-42 | **Different test** |
| 1. Lienhardt C, Fielding K, Hane AA, , et al. Evaluation of the prognostic value of IFN-gamma release assay and tuberculin skin test in household contacts of infectious tuberculosis cases in Senegal.PLoS One 2010;5:e10508. | **Different test** |
| 1. Wang SH, Powell DA, Nagaraja HN, et al. Evaluation of a modified interferon-gamma release assay for the diagnosis of latent tuberculosis infection in adult and paediatric populations that enables delayed processing.Scand J Infect Dis 2010;42:845-50 | **Different test** |
| 1. Mazurek M, Jereb J, Vernon A, et al. Updated guidelines for using Interferon Gamma Release Assays to detect Mycobacterium tuberculosis infection – United States 2010. MMWR 2010;25:59-125 | **Guidelines** |
| 1. Lighter J, Rigaud M, Huie M et al. Chemokine IP-10: an adjunct marker for latent tuberculosis infection in children. Int J Tuberc Lung Dis 2009;13:731-736. | **IGRA method did not meet the inclusion criteria.** |
| 1. Whittaker E, Gordon A, Kampmann B. Is IP-10 a better biomarker for active and latent tuberculosis in children than IFN-gamma? PlosOne 2008;3:e3901 | **IGRA method did not meet the inclusion criteria.** |
| 1. Ferrara G, Losi M, D’Amico R et al. Use in routine clinical practice of two commercial blood test diagnosis of infection with Mycobacterium tuberculosis: a prospective study. Lancet 2006;367:1328-1334 | **No distinction between adults and children** |
| 1. Dewan PK, Grinsdale J, Kawamura LM. Low sensitivity of a whole-blood interferon-gamma release assay for detection of active tuberculosis. Ciln Infect Dis 2007;44:69-73 | **No clear distinction between adults and children** |
| 1. Nienhaus A, Schablon A, Diel R. Interferon-gamma release assay for the diagnosis of latent TB infection-analysis of discordant results, when compared to the tuberculin skin test. PLoS ONE 2008;3:e2665 | **No clear distinction between adults and children** |
| 1. Shams H, Weis SE, Kulcar P *et al.* Enzyme-linked immunospot and tuberculin skin testing to detect latent tuberculosis infection. Am J Respir Crit Care med 2005;172:1161-1168 | **No clear distinction between adults and children** |
| 1. Pai M, Joshi R, Dogra S *et al.* T-cell assay conversions and reversions among household contacts of tuberculosis patients in rural India. Int J Tuberc Dis 2009;13:84-92 | **No distinction between adults and children** |
| 1. Burl S, Hill PC, Jeffries DJ *et al.* FOXP3 gene expression in a tuberculosis case contact study. Clin Exp Immunol 2007;149:117-122 | **No pertinent to query search** |
| 1. Chou CH, Hsu HL, Lee LN, Hsueh PR, Luh KT. Comparison of 2 interferon-gamma assays and Roche Cobas Amplicor Mycobacterium tuberculosis assay for rapid diagnosis of tuberculosis among patients with suspected tuberculosis in Taiwan. J Microbiol Immunol Infect 2009;42:251-7. | **Not pertinent to query search** |
| 1. Cobat A, gallant CJ, Simkin L, et al. High heritability of antimycobacterial immunity in an area of hyperendemicity for tuberculosis disease. J Infect Dis 2010;1:15-9 | **Not pertinent to query search** |
| 1. Dodd PJ, Milington KA, Ghani AC, et al. Interpreting tuberculin skin tests in a population with a high prevalence of HIV, tuberculosis, and nonspecific tuberculin tuberculin sensitivity. Am J Epidemiol 2010;171:1037-45 | **Not pertinent to query search** |
| 1. Eisenhut MParanjothy S, Abubakar I, Bracebridge S, Lilley M, Mulla R, Lack K, Chalkley D, McEvoy M. BCG vaccination reduces risk of infection with Mycobacterium tuberculosis as detected by gamma interferon release assay.Vaccine 2009;2:6116-20 | **Not pertinent to query search** |
| 1. Eriksen J, Chow JY, Mellis V, et al. Protective effect of BCG vaccination in a nursery outbreak in 2009: time to reconsider the vaccination threshold? Thorax 2010; Oct 28 [Epub ahead of print] | **Not pertinent to query search** |
| 1. LoBue PA, Enarson BA, Thoen TC. Tuberculosis in humans and its epidemiology, diagnosis and treatment in the United States. Int J Tuberc Lung Dis 2010;14:1226-32 | **Not pertinent to query search** |
| 1. Martineau AR, Leandro AC, Anderson ST,et al. Association between Gc genotype and susceptibility to TB is dependent on vitamin D status. Eur Respir J 2010;35:1106-12. | **Not pertinent to query search** |
| 1. Ruhwald M, Petersen J, Kofoed K *et al.* Improving T-cell assay for diagnosis of latent TB infection: potential of a diagnostic test based on IP-10. PLoS ONE 2008;3:e2858 | **Not pertinent to query search** |
| 1. Soares A, Govender L, Hughes J, et al. Novel application of Ki67 to quantify antigen-specific in vitro lymphoproliferation. J Immunol Methods 2010;362:43-50 | **Not pertinent to query search** |
| 1. Soysal A, Bahceciler N, Barlan I *et al.* Lack of an inverse association between tuberculosis infection and atopy: by a T-cell-based immune assay (RD1 ELISpot). Pediatr Allergy Immunol 2008;19:709-715 | **Not pertinent to query search** |
| 1. Scriba TJ, Tameris M, Mansoor N, Smit E, et al. Modified vaccinia Ankara-expressing Ag85A, a novel tuberculosis vaccine, is safe in adolescents and children, and induces polyfunctional CD4+ T cells.Eur J Immunol 2010;40:279-90. | **Not pertinent to query search** |
| 1. Tena-Coki NG, Scriba TJ, Peteni N, et al. CD4 and CD8 T-cell responses to mycobacterial antigens in African children. Am J Respir Crit Care Med 2010;182:120-9 | **Not pertinent to query search** |
| 1. Sallakci N, Coskun M, Berber Z *et al.* Interferon-gamma gene+874T-A polymorphism is associated with tuberculosis and gamma interferon response. Tuberculosis 2007;87:225-230 | **Not pertinent to query search, adult patients** |
| 1. Thomas MM, Hinks TS, Reghuraman S *et al.* Rapid diagnosis of Mycobacterium tuberculosis meningitis by enumeration of cerebrospinal fluid antigen-specific T-cells. Int J Tuberc Lung Dis 2008;93:200-203 | **Not pertinent to query search. IGRA method did not meet the inclusion criteria.** |
| 1. Amdekar YK. How to optimize current (available) diagnostic tests. Indian J Pediatr 2010 Nov 6. [Epub head of print] | **Review** |
| 1. Bocchino M, Bellofiore B, Matarese A *et al.* IFN-gamma release assay in tuberculosis management in selected high-risk populations. Expert Rev Mol Diagn 2009;9:165-177 | **Review** |
| 1. Brinza N, Mihaescu T. Diagnostic difficulties in pulmonary tuberculosis in children. Rev med Chir Soc Med Nat lasi 2007;111:65-69 | **Review** |
| 1. [Dheda K](http://www.ncbi.nlm.nih.gov/pubmed?term=), [Smit RZ](http://www.ncbi.nlm.nih.gov/pubmed?term=), [Badri M](http://www.ncbi.nlm.nih.gov/pubmed?term=), *et al.* T-cell interferon-gamma release assay for the rapid immunodiagnosis of tuberculosis: clinical utility in high-burden vs. low-burden settings. Curr Opin Pulm Med 2009;15:188-2009 | **Review** |
| 1. Higuchi K, Harada N, Fukazawa K *et al.* Relationship between whole-blood interferon-gamma responses and the risk of active tuberculosis. Tuberculosis 2008;88:244-248 | **Review** |
| 1. Lagrange PH, Simmonney, Herrmann JL. New immunological tests in the diagnosis of tuberculosis. Rev Mal Respir 2007;24:453-472 | **Review** |
| 1. Lalvani A, Pareek M. Interferon gamma release assays: principles and practice. Enferm Infecc Microbiol Clin 2010;28:245-52. | **Review** |
| 1. Lalvani A. MIlington KA. T-cell based diagnosis of childhood tuberculosis infection. Curr Opin Infect Dis 2007;264-271 | **Review** |
| 1. Lewinson DA, Lobato MN, Jereb JA, et al. Interferon-gamma release assays: new diagnostic tests for *Mycobacterium tuberculosis* infection and their use in children. Curr Opin Pediatr 2010;22:71-6. | **Review** |
| 1. Pottumarthy S, Morris AJ, Harrison AC *et al.* Evaluation of the tuberculin gamma interferon assay: potential to replace the Mantoux. J Clin MIcrobiol 1999;37:3229-3232 | **Review** |
| 1. Richeldi L. An update on the diagnosis of tuberculosis infection. Am J Resp Crit Care Med 2006;174:736-742 | **Review** |
| 1. Solovic I, Sester M, Gomez-Reino JJ, et al. The risk of tuberculosis related to tumour necrosis factor antagonist therapies: a TBNET consensus statement. Eur Respir J 2010;36:1185-206 | **Review** |
| 1. Zare HJ, Connell TG, Nicol M. Diagnosis of polmonary tuberculosis in children: new advances. Expert rev Anti Infect Ther 2010;8:277-88. | **Review** |
| 1. Banfield S, Pascoe E, Thambiran A, Siafarikas A, Burgner D. Factors associated with the performance of a blood-based interferon-γ release assay in diagnosing tuberculosis. PLoS One 2012;7:e38556. doi: 10.1371/journal.pone.0038556. Epub 2012 Jun 12. | **Not specified results for children and adult populations** |
| 1. Cassone A, Cauda R, De Maria A. High rate of Quantiferon positive and tuberculin negative tests in infants born at a large Italian university hospital in 2011: a cautionary hypothesis. Pathog Glob Health. 2012;106:8-11. | **Hypothesis on IGRA performance** |
| 1. Mandalakas AM, Detjen AK, Hesseling AC, Benedetti A, Menzies D. Interferon-gamma release assaysand childhood tuberculosis:systematic review and meta-analysis. Int J Lung Dis 2011;15:1018-32. | **Review** |
| 1. Sun L, Xiao J, Miao Q, Feng W, Wu X, Yin Q, Jiao W, Shen C, Liu F, Shen D, Shen A. Interferon gamma release assay in diagnosis of pediatric tuberculosis: a meta.analysis. FEMS Immunol Med Microbiol 2011;63:165-73. | **Review** |
| 1. Chen J, Zhang R, Wang J, Liu L, Zheng Y, Shen Y, Qi T, Lu H. Interferon-gamma release assays for the diagnosis of active tuberculosis in HIV-infected patients: a systematic review and meta-analysis. PLoS One 2011;6: e 26827. | **Review, adults only** |
| 1. Machingaidze S, Wisonge CS, Gonzalez-Angulo Y, Hatherill M, Moyo S, Hanekom W, Mahomed H. The utility of an interferon gamma release assay for diagnosis of latent tuberculosis infection and disease in children. A systematic review and meta-analysis. Pediatr Infect Dis J 2011;30:694-700. | **Review** |
| 1. Legesse M, Ameni G, Mamo G, Medhin G, Bjune G, Abebe F. Association of the level of IFN-γ produced by T cells in response to Mycobacterium tuberculosis-specific antigens with the size of skin test indurations among individuals with latent tuberculosis in a highly tuberculosis-endemic setting. Int Immunol 2012;24:71-8. | **Adult population** |
| 1. Okimoto N, Nanba F, Kurihara T, Miyashita N. The positive response rate with QuantiFERON-TB GOLD In-Tube in patients with Mycobacterium avium complex. Kekkaku. 2012 Apr;87(4):337-9. | **Not pertinent to the query search.** |
| 1. Simpson T, Fox J, Crouse K, Field K. Screening for Mycobacterium tuberculosis using an interferon-gamma release assay. J Public Health Manag Pract. 2012 Jul-Aug;18(4):E19-25. doi: 10.1097/PHH.0b013e31822adef1. | **Not pertinent to the query search.** |
| 1. Frieri M, Settipane RA. What every physician should know about rapid diagnosis of Mycobacterium tuberculosis infection in children using interferon-gamma release assays. Allergy Asthma Proc. 2012 May-Jun;33(3):215-6. doi: 10.2500/aap.2012.33.3580. | **Not pertinent to the query search.** |
| 1. Knappik M, Schönfeld N, Günther A, Bergmann T, Magdorf K, Rüssmann H, Mauch H, Barker M. Interferon-gamma release assays for hospital-based tuberculosis diagnostics in children and adolescents--a retrospective analysis. Pneumologie. 2012 Apr;66(4):207-11. doi: 10.1055/s-0032-1308914. Epub 2012 Apr 4. | **Article in German** |
| 1. Freeman JT, Marshall RJ, Newton S, Austin P, Taylor S, Chew TC, Gavaghan S, Roberts SA. Screening for Mycobacterium tuberculosis infection among healthcare workers in New Zealand: prospective comparison between the tuberculin skin test and the QuantiFERON-TB Gold In-Tube assay. N Z Med J. 2012 Feb 10;125(1349):21-9. | **Adult population** |
| 1. Komukai J, Matsumoto K, Tomihara A, Miyake Y, Tatsumi T, Arima K, Danno K, Hirota S, Yoshida H, Koda S, Terakawa K, Shimouchi A. Comparative performance of tuberculin skin test and QuantiFERON TB-gold in contact investigations for tuberculosis. Kekkaku. 2011 Nov;86(11):847-56. | **Article in Japanese** |
| 1. Moon HW, Kim H, Hur M, Yun YM, Lee A. Latent tuberculosis infection screening for laboratory personnel using interferon-γ release assay and tuberculin skin test in Korea: an intermediate incidence setting. J Clin Lab Anal. 2011 Nov;25(6):382-8. doi: 10.1002/jcla.20479. | **Adult population** |
| 1. Talebi-Taher M, Javad-Moosavi SA, Entezari AH, Shekarabi M, Parhizkar B. Comparing the performance of QuantiFERON-TB Gold and Mantoux test in detecting latent tuberculosis infection among Iranian health care workers. Int J Occup Med Environ Health. 2011 Dec;24(4):359-66. doi: 10.2478/s13382-011-0046-7. Epub 2011 Nov 16. | **Adult population** |
| 1. Elzi L, Steffen I, Furrer H, Fehr J, Cavassini M, Hirschel B, Hoffmann M, Bernasconi E, Bassetti S, Battegay M. [Improved sensitivity of an interferon-gamma release assay (T-SPOT.TB™) in combination with tuberculin skin test for the diagnosis of latent tuberculosis in the presence of HIV co-infection.](http://www.ncbi.nlm.nih.gov/pubmed/22085801) BMC Infect Dis. 2011 Nov 15;11:319. doi: 10.1186/1471-2334-11-319. | **Adult population** |
| 1. Nnaji GA, Chukwu J, Ugochukwu EF, Ezechukwu C, Ogbonnaya L. Diagnostic approach of childhood pulmonary tuberculosis in endemic areas of Southeast Nigeria. Niger J Med. 2011 Jul-Sep;20(3):327-32. | **Not pertinent to the query search** |
| 1. Soysal A, Bakir M.T-SPOT.TB assay usage in adults and children. Expert Rev Mol Diagn. 2011 Jul;11(6):643-60. doi: 10.1586/erm.11.46. | **Review** |
| 1. Delgado Naranjo J, Castells Carrillo C, García Calabuig MÁ, Sáez López I; en representación del Grupo de Trabajo para el estudio de los IGRAS en Bizkaia y Araba (GTIBA). Comparative performance of QuantiFERON(®)-TB Gold IT versus tuberculin skin test among contact investigations for latent tuberculosis infection. Med Clin (Barc). 2011 Sep 17;137(7):289-96. doi: 10.1016/j.medcli.2010.11.036. Epub 2011 Apr 27. | **Article in Spanish** |
| 1. Chiappini E, Accetta G, Bonsignori F, Boddi V, Galli L, Biggeri A, De Martino M. [Interferon-γ release assays for the diagnosis of Mycobacterium tuberculosis infection in children: a systematic review and meta-analysis.](http://www.ncbi.nlm.nih.gov/pubmed/23058005) Int J Immunopathol Pharmacol. 2012;25:557-64. | **Review** |
| 1. Chiappini E, Bonsignori F, Accetta G, Boddi V, Galli L, Biggeri A, De Martino M. [Interferon-γ release assays for the diagnosis of Mycobacterium tuberculosis infection in children: a literature review.](http://www.ncbi.nlm.nih.gov/pubmed/22697065) Int J Immunopathol Pharmacol 2012;25:335-43. | **Review** |
| 1. Kakkar F, Allen U, Ling D, Pai M, Kitai I. Tuberculosis in children: New diagnostic blood tests. Paediatr Child Health 2010;15:529-38. | **Review** |
| 1. Soysal A, Bakir M. T-SPOT.TB assay usage in adults and children. Expert Rev Mol Diagn. 2011;11:643-60. | **Review** |
| 1. Zellweger A, Zellweger JP. [Influence of pre-analytic conditions on the rate of indeterminate T-SPOT.TB tests.](http://www.ncbi.nlm.nih.gov/pubmed/21719500) Eur Respir J. 2011;38:221-2. | **Not pertinent to the query search** |
| 1. Ang M, Wanling W, Chee SP. Clinical significance of an equivocal interferon y release assay result. Br J Ophthalmol. 2012 ;96:284-8. | **Not pertinent to the query search** |
| 1. Talbot EA, Harland D, Wieland-Alter W, Burrer S, Adams LV. Specificity of the tuberculin skin test and the T-SPOT.TB assay among students in a low-tuberculosis incidence setting. J Am Coll Health. 2012;60(1):94-6. doi: 10.1080/07448481.2011.580029. | **Adults only** |
| 1. Chegou NN, Detjen AK, Thiart L, Walters E, Mandalakas AM, Hesseling AC, Walzl G. Utility of host markers detected in quantiferon supernatants for the diagnosis of tuberculosis in children in a high-burden setting. PLoS One 2013;8:e64226. | **Not pertinent** |
| 1. Pollock L, Basu Roy R, Kampmann B. How to use: interferon γ release assays for tuberculosis. Arch Dis Child Educ Pract Ed 2013;98:99-105. | **Review** |
| 1. Dhanasekaran S, Jenum S, Stavrum R, Ritz C, Faurholt-Jepsen D, Kenneth J, Vaz M, Grewal HM, Doherty TM; TB Trials Study Group, Doherty M, Grewal HM, Hesseling AC, Jacob A, Jahnsen F, Kenneth J, Kurpad AV, Lindtjorn B, Macaden R, Nelson J, Sumithra S, Vaz M, Walker R. Identification of biomarkers for Mycobacterium tuberculosis infection and disease in BCG-vaccinated young children in Southern India. Genes Immun 201. doi: 10.1038/gene.2013.26. | **Not pertinent** |
| 1. Gwee A, Pantazidou A, Ritz N, Tebruegge M, Connell TG, Cain T, Curtis N. To x-ray or not to x-ray? Screening asymptomatic children for pulmonary TB: a retrospective audit. Arch Dis Child 2013;98:401-4. | **Not pertinent** |
| 1. Rose MV, Kimaro G, Kroidl I, Hoelscher M, Bygbjerg IC, Mfinanga SM, Ravn P. Evaluation of QuantiFERON microtube, using 0.9 mL blood, for diagnosing tuberculosis infection. Eur Respir J 2013;41:909-16. | **Not pertinent** |
| 1. Chauhan S, Gahalaut P, Rathi AK. Tuberculin Skin Test, chest radiography and contact screening in children ≤5 y: relevance in Revised National Tuberculosis Control Programme (RNTCP). Indian J Pediatr 2013;80(4):276-80. | **Only TST was performed** |

**Appendix 3. Quality assessment of the studies included in the meta-analysis**

| **Study,**  **Year** | **Country** | **Population**  **(study design)** | **Children (n)** | **Age (mean; median; range)** | **Used IGRA** | **TST cut off values** | **Blind test** | **Interval from TST to IGRA** | **Criteria for diagnosis of Tb disease** | **Immunocompromised population** | **Follow-up** |
| --- | --- | --- | --- | --- | --- | --- | --- | --- | --- | --- | --- |
| **Altet-Gomez** **N**, **2010** | Spain | Children from contact-tracing studies and healthy controls with positive TST results  (Prospective study) | 166  (Contact group=98;  Screening group=68)) | Mean age 9.08±4.85 years | T-SPOT.TB  QFT-G-IT | ≥5 mm in contacts and in children  with abnormal chest radiographs consistent with active TB; and ≥10 mm for children in  the SG, irrespective of BCG immunisation. | yes | 5 days | B | NO | NO |
| **Banford AR, 2009** | **UK** | Suspected active TB cases (cross sectional) | 333 | Mean 8.5 yrs Range: 2mo-16 yrs | QFT-G-IT | 15 mm | Yes | Not reported | B | NO | NO |
| **Bianchi L, 2009** | Italy | Outpatients,  including  immigrants  and  internationall  y adopted  children and  inpatients  with  suspected TB  (crosssectional) | 336 | Median 54  mo  Range  31.2-82.7  mo | QFT-G-IT | >5 mm in  close  contact or  suspected  contagious  case of TB  disease or  children  suspected  to have TB  disease  > 10 mm in  children  born in  countries  with a high  prevalence  of TB and  recently  immigrated | Yes | Same day | B | No | No |
| **Connell**  **TG, 2008** | Australia | Suspected  active TB  cases and  high-risk of  latent TB  infection  cases  (cross –  sectional) | 100 | Median 7  yrs. Range  1-19 | QFT-G.IT  T-SPOT.TB | >10mm in  patients  with  moderate  risk factors  ( age 1-5  years;  origin from  high  prevalence  countries)  > 5 mm in  patients  with high  risk  (household  TB contact;  age less 1  year)  > 15 mm if  BGC  within 5  years and  moderate  risk | Yes | Same Day | B | No | No |
| **Detjen AK,**  **2007** | Germany | Inpatients  with  confirmed  TB; NTM  lymphadenitis  ; other  respiratory  tract  infections  (crosssectional) | 73 | Median 39  mo Range  1-5 yrs | QFT-G-IT  T-SPOT.TB | >5mm | Yes | Not reposrted | A | No (HIV+5%) | No |
| **Grare M,**  **2010** | France | Children at risk with TB (recent contact or recent immigration)or with suspected active TB disease | 51 | Healthy contacts (n=31)  mean age 5.8 years;  LTBI children (n=13) mean age 5.6  Active TB children (N=7) mean age 6.5 years | QFT G-IT | >15 mm positive results, >10 mm for suspicion of TB, > 5 mm for unspecific reaction due to BCG vaccination | Yes | Same day | B | NO  (1/51 child was immunosuppressed) | NO |
| **Hansted E**  **2009** | Lithuania | Bacteriologic  ally  confirmed TB  cases,  contacts with  a case of  infectious  pulmonary  TB and  children  without any  known risk  for TB  (cross  sectional) | 120 | Median  14.0 yrs | T-SPOT.TB | 10 mm | Yes | Same day | A | No | 8 weeks |
| **Herrmann**  **JL, 2009** | France | Latent TB  infection or  active TB  cases  (prospective) | 131 | Median  7.4 yrs.  Range 2  mo-17 yrs | QFT-G-IT | > 10 mm in  no BCG  vaccinated  or in BCG  vaccinated  with known | Yes | Some day | B | No | 6 months |
| **Kampmann**  **B, 2009** | UK | Active or  latent TB  cases  (crosssectional) | 209 | Mean 6.9  yrs  Range 0-16  yrs | QFT-G-IT  TSPOT.  T  B | Considered  two  different  cut-off  levels  >10mm or  >15mm | Yes | Not reported | B | NO | NO |
| **Tavast E,**  **2009** | Finland | Children at  risk for TB  infection  (retrospective) | 27 | Median 9  yrs  Range 0-18  yrs | QFT-G-IT  T-SPOT.TB | > 10 mm in  BCGvaccinated  children  > 5 mm in  nonvaccinated  children | Yes | Not reported | B | No | No |
| **Tsolia M , 2010** | Greece | Children at risk for TB infection  (Cross sectional) | 286 | aged <15 years | QFT-G-IT | >10 mm for BCG vaccinated children  > 5 for unvaccinated BCG children with active TB or positive contact story  and  >10 for unvaccinated children with other high risk factors | Yes | NR | B | NO | NO |
| **Bergamini BM, 2009** | Italy | Recent immigration or household contacts, or suspected pulmonary or lymph-nodal TB (retrospective study) | 496 | Median 11.1 ± 5.7 years (Range 0-19 years) | QFT-G  QFT-G-IT  T-SPOT-TB. | 5 mm suspected active TB or recent TB contact; 10 mm in children aged less than 4 years or recently immigrated from a TB-endemic area | Not reported | Not reported | B | NO | no |
| **Haustein T, 2009** | UK | Suspected active or latent TB cases  (high prevalence of immunodeficient patients) retrospective) | 269 | Median 7.3 yrs. (IQR 2.7-12.6) | QFT-G-IT | > 5 mm or >15 mm if BCG vaccinated | yes | Not reported | B | 59 (25%) immunocompromised | yes |
| **Liebeschuetz S, 2004** | South Africa | Suspected TB cases (prospective) | 293 | Median 50 months (IQR: 22-84 mo) | In house ELISPOT (ESAT-6 and CFP-10) | > 0 mm in HIV-1 infected and > 15 mm in other children | yes | Same day | B | 75 HIV+ children/164 tested (46%) | no |
| **Okada K, 2008** | Cambodia | Household contacts of TB cases (cross-sectional) | 195 | Range: 0-5 yrs | QFT-G | > 10 mm | Not reported | Not reported | B | NO | Not reported |
| **Pavic C, 2011** | Croatia | BCG-vaccinatedchidren with History of exposure to active TB | 142 | Aged ≤5 years | QFT-G-IT | ≥10 mm | Not specified | Same day | - | NO | yes |
| **Mendez-Echevarria, 2011** | Spain | Immunocompetent children aged less than 15 years, who immigrated from TB endemic areas in the prior 2 years, children who were in contact with active TB, and children admitted with symptoms suggestive of TB (trasversal multicenter study) | 459 | Aged 1 month-15 years | QFT-G-IT | ≥10mm in immigrant children screened; ≥5mm in children in contact with active TB or with symptoms/radiological studies/pathological studies compatible with TB | yes | Not reported | B | NO | NO |
| **Thomas B, 2011** | UK | Immunocompetent children contacts of adult patients with active TB | 283 | 0-16 years; mean age 5.3 (SD 4.1) years | QFT-G-IT | ≥6 mm for unvaccinated; ≥15 mm for BCG vaccinated children | yes | Not reported | B | NO | NO |
| **Markova R, 2011** | Bulgaria | Children admitted with clinical or radiological features compatible with active TB | 68 | 0-16 years | QFT-G-IT | ≥15 mm | Not reported | Not reported | B | No exclusion criteria | NO |
| **Mahomed H, 2011** | South Africa | Adolescents recruited from high schools in the study area | 5244 | 12-18 years | QFT-G-IT | ≥5 mm | Not reported | Same day | B | No exclusion criteria | yes |
| **Dayal R, 2011** | India | Evaluation of 82 cases of active TB (68 pulmonary cases and 14 central-nervous.system cases); 48 disease matched controls and 20 TST+ controls (prospective) | 150 | <18 years | QFT-G-IT | Not reported | Not reported | Not reported | B | NO | yes |
| **Debord C, 2011** | France | Retrospective analysis of 19 immunocompetent French children with active TB | 19 | <6 years (range: 0.29-5.36 years; median: 1.52) | QFT-G-IT | ≥5 mm for unvaccinated; ≥10 mm for BCG vaccinated children | Yes | Not reported | B | NO | NO |
| **Losi M, 2011** | Italy | Screening of immigrant children and adolescents for the diagnosis of LTBI (perpective study) | 621 children screened with TST; in the 232 with positive or borderline result QFT-G-IT was perfomed | < 19 years | QFT-G-IT | 5-9 mm considered borderline; ≥10 mm considered positive | Not specified, 100% of TB cases | 31±29 days | A | NO | yes |
| **Moyo S, 2011** | South Africa | Children evaluated for TB disease in a high TB incidence setting (perpective study) | 400 children enrolled; 397 with both TST and QFT –G-IT results | < 3 years (median age 23 months; range 9-34 months) | QFT-G-IT | ≥10 mm | yes | Not reported | B | 2 (0.5%) children were HIV +; neither had TB disease | yes |
| **Kasambira TS, 2011** | South Africa | Cross-sectional study with limited longitudinal follow-up in children household contacts of adults with newly diagnosed pulmonary TB | 270 | Range: 6 months-16 years | QFT-G-IT | ≥ 5 mm | Not reported | Same day | B | 14/270 HIV infected (5%) | yes |
| **Shah M, 2011** | South Africa | Prospective study with 6 months longitudinal follow-up of children household contacts of adults with newly diagnosed pulmonary TB previously evaluated | 196 | Range: 6 months-16 years; median age 6 years (IQR:3-9years) | QFT-G-IT | ≥ 5 mm | Not reported | Same day | B | 7/196 HIV infected (4%) | yes |
| **Rutherford ME, 2012** | Indonesia | Evaluation of children exposed to sputum smear and chest-X-ray positive adult TB patients at household and neighbourhood level, recrited in a community-based lung clinic (prospective) | 299 household-exposed and 72 neighborhood exposed children | Range: 6 months-9 years; Median age: 61 months | QFT-G-IT | ≥ 10 mm | Not reported | Not reported | B | Not evaluated | yes |
| **Riazi S, 2012** | USA | Children evaluated because of an ambiguous diagnostically interpretable TST, referred mainly by pediatricians from private and public health clinics (prospective) | 517 | Range: 1 month-18 years | QFT-G | ≥ 10 mm | Not reported | Not reported | B | NO | yes |
| **Yassin MA, 2011** | Ethiopia | Evaluation of children with symtoms of TB, children in contact with adults with pulmonary TB and community controls (Cross-sectional study) | 322 children with symtoms of TB, 335 children in contact with adults with pulmonary TB and 156 community controls | Range: 1-15 years; median age 5-8 years | QFT-G-IT | ≥ 10 mm | Not reported | Not reported | B | 52 HIV positive children on 641 totals tested (8.11%) | yes |
| **Basu Roy R, 2012** | Greece, Spain, Italy, Bulgaria, UK | Children referred for evaluation of LTBI who had received both TST and one IGRA as part of the TB screening process in their setting (retrospective analysis) | 1128 | Range: 0-16 years | QFT-G-IT  T-SPOT.TB | ≥ 10 mm | Not reported | Simultaneously or at least within 7 days | B | NO | yes |
| **Cruz AT, 2011** | USA | Children with or without risk factors for TB evaluated in 3 tuberculosis clinics (prospective study) | 210 | Range: 1 mo. – 18 years; median age: 8.6 years | T-SPOT.TB | ≥ 15 mm in children without risk factors;  ≥ 10 mm for children with chronic medical problems or exposure to people at high risk; ≥ 5 mm for children with suspected disease/immunocompromised or with identifiable source case | yes | Simultaneously in 18 cases, TST performed months previously in the other children | B | NO | yes |
| **Stavri HR, 2010** | Romania | Hospitalized children with TB | 60 | Range: 1-18 years; mean age: 9.44 ± 5.27 | QFT-G-IT | ≥ 10 mm | yes | Same day | B | NO | yes |
| **Mandalakas AM, 2012** | South Africa | South –african children presenting for out-patient care | 250 | Range: 3 months-15 years | QFT-G-IT; T-SPOT.TB | ≥ 5 mm in HIV-infected children; ≥ 10 mm in others | Yes | Same day | B | 130 HIV-infected children (52%) | yes |
| **Rose MV, 2012** | Tanzania | Children with sign and symptoms of TB (prospective) | 211 | Range: 0-15 years; mean age: 4.4 ± 3.8) | QFT-G-IT | ≥ 5 mm in HIV-infected children; ≥ 10 mm in others | Yes | Not reported | B | 78 HIV-infected children (37%) | yes |
| **Nkurunungi G, 2012** | Uganda | Children evaluated for LTBI (cross-sectional, observational study) | 907 | Aged 5 years | T-SPOT.TB | ≥ 10 mm | Yes | Same day and 3 weeks before | B | 13 HIV-infected (1.4%) | yes |
| **Nenadic N, 2011** | Croatia | Children exposed to active TB and/or with positive TST (prospective) | 59 | Range: 4-18 years; mean age: 12±4 years | QFT-G-IT | ≥ 10 mm | Not reported | Not reported | B | NO | yes |
| **Chiappini E, 2012** | Italy | Children diagnosed with LTBI or active TB (prospective) | 44 | Range :0-18 years | QFT-G-IT;  T-SPOT.TB | ≥ 5 mm in children in children in close contact with known or suspected contagious case of TB disease or for children suspected to have TB disease based on clinical evidence and/or chest radiograph; ≥ 10 mm for children born in countries with a high prevalence of TB and who recently immigrated | Not reported | Same day | B | NO | yes |
| **Chiappini E, 2012** | Italy | Children with clinical suspicion of TB disease and/or in close contact with recently diagnosed cases of contagious TB disease and/or internationally adopted or recently immigrated children coming from countries with a high prevalence of TB | 75 | Range:0-9 years | QFT-G-IT;  IFN-gamma and IL-2 ELISPOT | ≥ 5 mm in children in close contact with known or suspected contagious case of TB disease or for children suspected to have TB disease based on clinical evidence and/or chest radiograph; ≥ 10 mm for children born in countries with a high prevalence of TB and who recently immigrated | yes | Same day | B | NO | yes |
| **Connell TG, 2006** | South Africa (Australian author) | Suspected active TB cases and latent TB cases (cross-sectional) | 106 | Median age in children with LTB: 9,2 yrs; active TB: 3.9 yrs | QFT-G | >15 mm in BCG vaccinated, >5 mm in known TB contacts, >10 mm for all others | Not recorded | Not recorded | B | NO | No |
| **Chun JK, 2008** | Korea | Close TB contacts, casual TB contacts and control children (cross-sectional) | 227 | Range 0-15,8 yrs (median age 3,2 years) | QFT-G-IT | ≥ 5mm | Not recorded | Not recorded | B | NO | yes |
| **Dominguez J, 2008** | Spain | Active TB cases, or TB contacts or enrolled cases for screening of LTBI  (prospective evaluation) | 134 | Range 0-18 yrs | QFT-G-IT  T-SPOT.TB | > 5 mm | Not recorded | Not recorded | B | NO | No |
| **Sun L, 2010** | China | Children with active TB and non-tubercolous children (cross-sectional) | 125 | Mean age 7,1 years (SD 4,5) | TSPOT | >10 mm | Not reported | Not reported | B | NO | yes |
| **Soysal A, 2008** | Turkey | Healthy children aged between 6 and 10 years recruited during school usual TST screening before the second dose of BCG | 209 | Mean age 8.4 (SD 0.68) years | T-SPOT.TB | ≥ 15 mm | Not reported | Not reported | No active TB diagnosis | NO | no |
| **Dogra S, 2007 (31)** | India | Hospitalised cases with suspected TB disease or TB contacts (cross-sectional) | 105 | Median age 6 years (Range 1-12 years) | QFT-G-IT | > 10 mm | yes | Same day | B | NO | yes |
| **Lighter J, 2009 (41)** | USA | Children recruited from paediatric chest clinic, well-child clinic and paediatric inpatients ward (prospective) | 207 | Mean age 9 years (Range 0-18 years) | QFT-G-IT | 10 mm | yes | Same day | B | NO | no |
| **Noorbakhsh S, 2011** | Iran | Young household contacts of immunocompetent cases of proven pulmonary tuberculosis (cross-sectional study) | 59 | Aged less than 20 years | QFT-G | ≥ 10 mm | Not reported | Same day | B | NO | yes |
| **Carvalho, 2013** | Italy | Children contacts of a smear positive, drug-sensitive cavitary pulmonary TB in a hematology-oncology service (prospective study) | 18 | Median age 5,5 years (range: 1-18 years) | QFT-G-IT; T-SPOT.TB | Not stated | Not reported | Offered obnly in 5 cases, not stated with which interval | B | Yes (100%) | Yes |
| **Uluk, 2013** | Papua Guinea | Children with suspected TB (prospective, cross-sectional study) | 216 | Range: 1 month-12 years | QFT-G-IT | > 10 mm in BCG-vaccinated children; > 5 mm in not-vaccinated children | Yes | Same day | B | Yes (12,5%) | No |
| **Ling, 2013** | South Africa | Hospitalized children with suspected pulmonary TB | 557 | Median age 22 months (IQR 12-53) | T-SPOT.TB | > 5 mm in HIV-infected children,  > 10 mm in other children | Yes | Same day | B | Yes (24%) | No |

Appendix 4. Individual-study and pooled estimates for sensitivity, specificity, and summary of the study characteristics.

| Test | Author | Year | Country | Reference | % BCG | TP | FP | FN | TN | Sensitivity* | 95% CI |  | Specificity* | 95%CI | |
| --- | --- | --- | --- | --- | --- | --- | --- | --- | --- | --- | --- | --- | --- | --- | --- |
| T-SPOT.TB | ALTET-GOMEZ | 2010 | SPAIN | 59 | 70 | 11 | 0 | 2 | 27 | 0.821 | 0.531 | 0.969 | 0.982 | 0.845 | 1.000 |
| T-SPOT.TB | BAMFORD | 2009 | UNITED KINGDOM | 21 | 53 | 47 | 0 | 47 | 0 | 0.500 | 0.396 | 0.604 | - | - | - |
| T-SPOT.TB | CONNELL | 2008 | AUSTRALIA | 28 | 47 | 9 | 1 | 0 | 38 | 0.950 | 0.619 | 1.000 | 0.962 | 0.849 | 0.997 |
| T-SPOT.TB | DETJEN | 2007 | GERMANY | 30 | 5 | 26 | 1 | 2 | 39 | 0.914 | 0.749 | 0.985 | 0.963 | 0.853 | 0.997 |
| T-SPOT.TB | HANSTED | 2009 | LITHUANIA | 34 | 100 | 23 | 0 | 0 | 0 | 0.979 | 0.821 | 1.000 | - | - | - |
| T-SPOT.TB | KAMPMANN | 2009 | UNITED KINGDOM | 11 | 60 | 31 | 3 | 26 | 22 | 0.543 | 0.407 | 0.675 | 0.865 | 0.675 | 0.966 |
| T-SPOT.TB | NICOL | 2009 | SOUTH AFRICA | 46 | 100 | 23 | 8 | 35 | 42 | 0.398 | 0.273 | 0.534 | 0.833 | 0.703 | 0.923 |
| T-SPOT.TB | TAVAST | 2009 | FINLAND | 53 | 69 | 9 | 0 | 1 | 72 | 0.864 | 0.533 | 0.990 | 0.993 | 0.938 | 1.000 |
| T-SPOT.TB | WARIER | 2009 | INDIA | 57 | 8 | 41 | 1 | 14 | 46 | 0.741 | 0.607 | 0.849 | 0.969 | 0.873 | 0.998 |
| T-SPOT.TB | CRUZ | 2011 | USA |  | 10.47 | 23 | 0 | 7 | 72 | 0.741 | - | - | 0.972 | - | - |
| T-SPOT.TB | CHIAPPINI | 2012 | ITALY |  | - | 23 | - | 3 | - | 0.884 | 0.771 | 1.000 | - | - | - |
| T-SPOT.TB | SUN | 2010 | CHINA |  | 77.6 | 66 | 2 | 8 | 48 | 0.892 | - | - | 1.000 | - | - |
| T-SPOT.TB | DOMINGUEZ | 2008 | SPAIN |  | 64.17 | 6 | - | 1 | 0 | 0.667 | - | - | - | - | - |
| T-SPOT.TB | LING | 2013 | SOUTH AFRICA |  | 85 | 157 | - | 139 | - | 0.535 | - | - | - | - | - |
| QFT-G-IT | ALTET-GOMEZ | 2010 | SPAIN | 59 | 70 | 9 | 0 | 5 | 27 | 0.633 | 0.353 | 0.860 | 0.982 | 0.845 | 1.000 |
| QFT-G-IT | BAMFORD | 2009 | UNITED KINGDOM | 21 | 53 | 101 | 0 | 56 | 0 | 0.642 | 0.562 | 0.717 | - | - | - |
| QFT-G-IT | BIANCHI | 2009 | ITALY | 22 | 52 | 15 | 15 | 1 | 251 | 0.912 | 0.673 | 0.994 | 0.942 | 0.907 | 0.967 |
| QFT-G-IT | CONNELL | 2008 | AUSTRALIA | 28 | 47 | 8 | 2 | 1 | 44 | 0.850 | 0.497 | 0.989 | 0.947 | 0.839 | 0.991 |
| QFT-G-IT | DETJEN | 2007 | GERMANY | 30 | 5 | 26 | 0 | 2 | 40 | 0.914 | 0.749 | 0.985 | 0.988 | 0.892 | 1.000 |
| QFT-G-IT | GRARE | 2010 | FRANCE | 63 | 43 | 3 | 0 | 2 | 28 | 0.583 | 0.167 | 0.923 | 0.983 | 0.850 | 1.000 |
| QFT-G-IT | HERRMANN | 2009 | FRANCE | 35 | 91 | 25 | 1 | 7 | 11 | 0.773 | 0.594 | 0.900 | 0.885 | 0.591 | 0.992 |
| QFT-G-IT | KAMPMANN | 2009 | UNITED KINGDOM | 11 | 60 | 40 | 2 | 23 | 22 | 0.633 | 0.503 | 0.750 | 0.900 | 0.713 | 0.983 |
| QFT-G-IT | TAVAST | 2009 | FINLAND | 53 | 69 | 10 | 0 | 0 | 0 | 0.955 | 0.647 | 1.000 | - | - | - |
| QFT-G-IT | TSOLIA | 2010 | GREECE | 68 | 49 | 31 | 4 | 3 | 37 | 0.900 | 0.751 | 0.975 | 0.893 | 0.759 | 0.967 |
| QFT- G-IT | MENDEZ-ECHEVARRIA | 2011 | SPAIN |  | 47 | 61 | 3 | 1 | 304 | 0.983 | 0.940 | 1.000 | 0.970 | 0.970 | 1.000 |
| QFT-G-IT | THOMAS B | 2011 | UNITED KINGDOM |  | 71.7 | 2 | 0 | 0 | 236 | 1.000 | - | - | 0.971 | - | - |
| QFT-G-IT | MARKOVA R | 2011 | BULGARIA |  | 100 | 33 | 0 | 17 | 18 | 0.660 | - | - | 1.000 | - | - |
| QFT-G-IT | DAYAL R | 2011 | INDIA |  | 33.0 in cases; 75.0 in controls | 42 | 14 | 26 | 23 | 0.512 | - | - | 0.480 | - | - |
| QFT-G-IT | DEBORD C | 2011 | FRANCE |  | 84.2 | 15 | 0 | 4 | 0 | 0.789 | 0.600 | 1.000 | - | - | - |
| QFT-G-IT | LOSI M | 2011 | ITALY |  | Not specified, 100% of TB cases | 4 | - | 0 | - | 1.000 | - | - | - | - | - |
| QFT-G-IT | MOYO S | 2011 | SOUTH AFRICA |  | 100 | 20 | 48 | 29 | 279 | 0.380 | 0.250 | 0.530 | 0.810 | 0.760 | 0.850 |
| QFT-G-IT | KASAMBIRA TS | 2011 | SOUTH AFRICA |  | 95 | 29 | - | 8 | - | 0.783 | - | - | - | - | - |
| QFT-G-IT | YASSIN AM | 2011 | ETHIOPIA |  | 67.9 | 63 | 20 | 37 | 108 | 0.463 | - | - | 0.692 | - | - |
| QFT-G-IT | STAVRI HR | 2010 | ROMANIA |  | 100 | 27 | - | 15 | - | 0.450 | - | - | - | - | - |
| QFT-G-IT | ROSE MV | 2012 | TANZANIA |  | 93 | 5 | 7 | 22 | 61 | 0.180 | - | - | 0.897 | - | - |
| QFT-G-IT | NENADIC N | 2011 | CROATIA |  | 100 | 16 | - | 2 | - | 0.890 | - | - | - | - | - |
| QFT-G-IT | CHIAPPINI E | 2012 | ITALY |  | - | 24 | - | 2 | - | 0.923 | 0.826 | 1.000 | - | - | - |
| QFT-G-IT | CHIAPPINI E | 2012 | ITALY |  | 17.33 | 17 | 0 | 8 | 29 | 0.680 | - | - | 1.000 | - | - |
| QFT-G-IT | CHUN | 2006 | KOREA |  | 100 | 4 | - | 1 | - | 0.800 | - | - | - | - | - |
| QFT-G-IT | DOMINGUEZ | 2008 | SPAIN |  | 64.17 | 6 | - | 3 | - | 0.667 | - | - | - | - | - |
| QFT-G-IT | DOGRA | 2007 | INDIA |  | 92 | 7 | - | 4 | - | 0.640 | - | - | - | - | - |
| QFT-G-IT | LIGHTER | 2009 | USA |  | 36 | 3 | - | 0 | - | 1.000 | - | - | - | - | - |
| QFT-G-IT | HAUSTEIN | 2009 | UK |  | 51 | 21 | - | - | - | 0.778 | 0.573 | 0.901 | - | - | - |
| QFT-G-IT | OKADA | 2008 | CAMBODIA |  | 88 | 10 | - | - | - | 0.530 | - | - | - | - | - |
| QFT-G-IT | ULUK | 2013 | PAPUA GUINEA |  | 75 | 41 | 2 | 54 | 18 | 0.430 |  |  | 0.900 |  |  |
|  |  |  |  |  |  |  |  |  |  |  |  |  |  |  |  |
| TST | BAMFORD | 2009 | UNITED KINGDOM | 21 | 53 | 108 | 0 | 64 | 0 | 0.627 | 0.551 | 0.699 | - | - | - |
| TST | BIANCHI | 2009 | ITALY | 22 | 52 | 14 | 23 | 2 | 253 | 0.853 | 0.600 | 0.975 | 0.915 | 0.876 | 0.945 |
| TST | CONNELL | 2008 | AUSTRALIA | 28 | 47 | 7 | 1 | 2 | 48 | 0.750 | 0.394 | 0.956 | 0.970 | 0.878 | 0.998 |
| TST | DETJEN | 2007 | GERMANY | 30 | 5 | 28 | 27 | 0 | 18 | 0.983 | 0.850 | 1.000 | 0.402 | 0.260 | 0.557 |
| TST | GRARE | 2010 | FRANCE | 63 | 43 | 4 | 3 | 0 | 28 | 0.900 | 0.371 | 1.000 | 0.891 | 0.730 | 0.973 |
| TST | HANSTED | 2009 | LITHUANIA | 34 | 100 | 23 | 0 | 0 | 0 | 0.979 | 0.821 | 1.000 | - | - | - |
| TST | HERRMANN | 2009 | FRANCE | 35 | 91 | 27 | 5 | 4 | 7 | 0.859 | 0.691 | 0.956 | 0.577 | 0.283 | 0.835 |
| TST | KAMPMANN | 2009 | UNITED KINGDOM | 11 | 60 | 45 | 5 | 18 | 23 | 0.711 | 0.584 | 0.817 | 0.810 | 0.622 | 0.931 |
| TST | NICOL | 2009 | SOUTH AFRICA | 46 | 100 | 30 | 8 | 28 | 42 | 0.517 | 0.383 | 0.649 | 0.833 | 0.703 | 0.923 |
| TST | TAVAST | 2009 | FINLAND | 53 | 69 | 9 | 2 | 0 | 74 | 0.950 | 0.619 | 1.000 | 0.968 | 0.900 | 0.995 |
| TST | TSOLIA | 2010 | GREECE | 68 | 49 | 35 | 0 | 2 | 0 | 0.934 | 0.804 | 0.989 | - | - | - |
| TST | THOMAS | 2011 | UNITED KINGDOM |  | 71.7 | 2 | 6 | 0 | 237 | 1.000 | - | - | 0.975 | - | - |
| TST | MARKOVA | 2011 | BULGARIA |  | 100 | 41 | 0 | 3 | 18 | 0.820 | - | - | 1.000 | - | - |
| TST | DAYAL | 2011 | INDIA |  | 33.0 in cases; 75.0 in controls | 69 | 0 | 13 | 48 | 0.841 | - | - | 1.000 | - | - |
| TST | DEBORD | 2011 | FRANCE |  | 84.2 | 17 | - | 2 | - | 0.894 | - | - | - | - | - |
| TST | LOSI | 2011 | ITALY |  | Not specified, 100% of TB cases | 4 | - | 0 | - | 1.000 | - | - | - | - | - |
| TST | MOYO | 2011 | SOUTH AFRICA |  | 100 | 20 | 50 | 32 | 325 | 0.350 | 0.220 | 0.490 | 0.840 | 0.800 | 0.880 |
| TST | KASAMBIRA | 2011 | SOUTH AFRICA |  | 95 | 27 | - | 9 | - | 0.750 | - | - | - | - | - |
| TST | YASSIN | 2011 | ETHIOPIA |  | 67.9 | 102 | 20 | 51 | 121 | 0.750 | - | - | 0.775 | - | - |
| TST | CRUZ | 2011 | USA |  | 10.47 | 26 | 0 | 5 | 74 | 0.838 | - | - | 1.000 | - | - |
| TST (PPD-IC65) | STAVRI | 2010 | ROMANIA |  | 100 | 58 | - | 2 | - | 0.967 | - | - | - | - | - |
| TST (PPD-RT23) | STAVRI | 2010 | ROMANIA |  | 100 | 59 | - | 1 | - | 0.983 | - | - | - | - | - |
| TST | ROSE | 2012 | TANZANIA |  | 93 | 2 | 3 | 23 | 86 | 0.060 | - | - | 0.966 | - | - |
| TST | NENADIC | 2011 | CROATIA |  | 100 | 15 | - | 3 | - | 0.830 | - | - | - | - | - |
| TST | CHIAPPINI | 2012 | ITALY |  | 17.33 | 24 | 1 | 1 | 28 | 0.960 | - | - | 0.965 | - | - |
| TST | SUN | 2010 | CHINA |  | 77.6 | 57 | 0 | 17 | 36 | 0.770 | - | - | 1.000 | - | - |
| TST | CHUN | 2008 | KOREA |  | 100 | 3 | - | 2 | - | 0.600 | - | - | - | - | - |
| TST | DOMINGUEZ | 2008 | SPAIN |  | 64,17 | 9 | - | 0 | - | 1.000 | - | - | - | - | - |
| TST | DOGRA | 2007 | INDIA |  | 92 | 9 | - | 2 | - | 0.820 | - | - | - | - | - |
| TST | LIGHTER | 2009 | USA |  | 36 | 2 | - | 1 | - | 0.660 | - | - | - | - | - |
| TST | HAUSTEIN | 2009 | UK |  | 51 | 20 | - | 7 | - | 0.720 | 0.504 | 0.871 | - | - | - |
| TST | OKADA | 2008 | CAMBODIA |  | 88 | 15 | - | - | - | 0.790 | - | - | - | - | - |
| TST | ULUK | 2013 | PAPUA GUINEA |  | 75 | 29 | 0 | 73 | 15 | 0.225 | - | - | 1.000 | - | - |
| TST | LING | 2013 | SOUTH AFRICA |  | 85 | 193 | - | 103 | - | 0.652 | - | - | - | - | - |

Legend: BCG: Bacille Calmette-Guérin vaccine; IGRA: interferon-γ release assay;; QFT-G-IT: QuantiFERON®-TB Gold In-Tube; TST: tuberculin skin test; 95%CI: 95% confidence interval; §Positive cases/total of children with tuberculosis disease; ¶ Negative cases/total of healthy children without tuberculosis infection. * We added 0.5 to each cell of studies. Cox DR. The analysis of binary data. London: Methuen; 1970~~.~~

**Appendix 5.** T-SPOT.TB®, QuantiFERON®-TB Gold In tube and tuberculin skin test data in microbiologically confirmed active tuberculosis cases among the studies included in the sub-analysis to calculate meta-analytic estimates for sensitivity.

| **Author (Reference)** | **Year** | **Country** |  | **Positive (n/N)** |
| --- | --- | --- | --- | --- |
| **T-SPOT.TB** | |  |  |  |
| BAMFORD | 2009 | UNITED KINGDOM | 18/26 | |
| DETJEN | 2007 | GERMANY | 26/28 | |
| HANSTED | 2009 | LITHUANIA | 23/23 | |
| KAMPMANN | 2009 | UNITED KINGDOM | 14/22 | |
| NICOL | 2009 | SOUTH AFRICA | 5/10 | |
| WARIER  CRUZ  SUN  LING | 2009  2011  2010  2013 | INDIA  USA  CHINA  SOUTH AFRICA |  | 8/15  12/13  15/18  75/91 |
| **QFT-G-IT** |  |  |  |  |
| BAMFORD | 2009 | UNITED KINGDOM | 36/42 | |
| BIANCHI | 2009 | ITALY |  | 6/6 |
| DETJEN | 2007 | GERMANY | 26/28 | |
| GRARE | 2010 | FRANCE |  | 3/3 |
| KAMPMANN | 2009 | UNITED KINGDOM | 20/22 | |
| TSOLIA  MENDEZ-ECHEVARRIA  MARKOVA  DAYAL  DEBORD  LOSI  YASSIN  ROSE  CHIAPPINI  HAUSTEIN  DOGRA | 2010  2011  2011  2011  2011  2011  2011  2012  2012  2009  2007 | GREECE  SPAIN  BULGARIA  INDIA  FRANCE  ITALY  ETHIOPIA  TANZANIA  ITALY  UK  INDIA |  | 11/11  61/68  13/17  20/26  6/8  4/4  16/28  1/4  0/5  12/16  3/5 |
| **TST** |  |  |  |  |
| BAMFORD | 2009 | UNITED KINGDOM | 45/49 | |
| BIANCHI | 2009 | ITALY |  | 5/6 |
| DETJEN | 2007 | GERMANY | 28/28 | |
| GRARE | 2010 | FRANCE |  | 3/3 |
| HANSTED | 2009 | LITHUANIA | 23/23 | |
| KAMPMANN | 2009 | UNITED KINGDOM | 20/25 | |
| NICOL | 2009 | SOUTH AFRICA | 8/18 | |
| TSOLIA | 2010 | GREECE |  | 11/13 |
| MARKOVA | 2011 | BULGARIA |  | 14/17 |
| LOSI | 2011 | ITALY |  | 4/4 |
| YASSIN | 2011 | ETHIOPIA |  | 22/28 |
| CRUZ AT | 2011 | USA |  | 10/13 |
| CHIAPPINI E | 2012 | ITALY |  | 4/5 |
| SUN L | 2010 | CHINA |  | 11/18 |
| HAUSTEIN T | 2009 | UK |  | 10/16 |
| DOGRA S | 2007 | INDIA |  | 3/5 |
| LING | 2013 | SOUTH AFRICA |  | 68/91 |

Note. QFT-G-IT: QuantiFERON®-TB Gold *In-Tube;* TST: tuberculin skin test

**Appendix 6.** Summary of results from relevant studies on Interferon-γ release assays (IGRAs) in children

| **Author, Year** | **Country** | **Population**  **(study design)** | **Children (n)** | **Age (mean; median; range)** | **Used IGRA** | **BCG –vaccinated children (%)** | **indeterminate results (%)** | **TST cut-off values** | **Agreement between TST and IGRA (k)** | **Agreement between QFT and ELISPOT (k)** |
| --- | --- | --- | --- | --- | --- | --- | --- | --- | --- | --- |
| **Adetifa I,** **2010** | Gambia | Household contacts of newly diagnosed TB children | 285 | Range: 6 months - 14 years | T-SPOT.TB  QFT G-IT | 59.1% | T-SPOT.TB :0%  QFT G-IT : 0.8% | > 10 mm | T-SPOT.TB: K=0.54  QFT-G-IT**:** K =0.52 | K =0.55 |
| **Altet-Gomez N,** **2010** | Spain | TB contacts (CG) and controls with positive TST (SG) (Prospective study) | 166 | Mean age 9.08±4.85 years | T-SPOT.TB  QFT-G-IT | 69.9% | T-SPOT.TB: 1.2%  QFT-G-IT: 0.6% | >5 mm in contacts and in children with abnormal chest X Ray  >10 mm for children in the SG. | *BCG immunized :*  QFN-G-IT: K = 0.087  T-SPOT.TB K = 0.095  *Not BCG immunized*:  QFN-G-IT: K = 0.844  T-SPOT.TB: K = 0.887 | Not calculated |
| **Bakir M, 2009** | Turkey | TB contacts  (cross-sectional) | 979 | Mean 7 yrs; Range 1 mo-16 yrs | In house ELISPOT  (ESAT-6; CFP-10) | 67% | NR | 10 mm | Not calculated | Not calculated |
| **Bakir M,** **2009** | Turkey | TB contacts (prospective) | 908 | Mean 7 yrs; Range 1 mo-16 yrs | In house ELISPOT (ESAT-6; CFP-10) | 80% | NR | 10 mm | Not calculated | Not calculated |
| **Bamford AR, 2009** | UK | Suspected active TB cases (cross-sectional) | 333 | Mean 8.5 yrs (2mo-16 yrs) | QFT-G-IT  T-SPOT.TB | 53.3 % | 8% | 15 mm | T-SPOT.TB : K =-015  QFT-G-IT : K = 0.41 | Not calculated |
| **Basu Roy R, 2012** | Greece, Spain, Italy, Bulgaria, UK | Children referred for evaluation of LTBI who had received both TST and one IGRA as part of the TB screening process in their setting (retrospective analysis) | 1128 | Range: 0-16 years | QFT-G-IT  T-SPOT.TB | 61.7% | QFT-G-IT: 1.8%  T-SPOT.TB: 1.6% | ≥10 mm | QFT-G-IT: K= 0.23  T-SPOT.TB: K=0.35  Agreement between QFT-G-IT and T-SPOT.TB: K=0.73 | Not calculated |
| **Bianchi L,** **2009** | Italy | Outpatients, including immigrants and internationally adopted children and inpatients with suspected TB(cross-sectional) | 336 | Median 54 mo ( 31.2-82.7 mo) | QFT-G-IT | 51.5% | 0.6% | >5 mm in close contacts or suspected active TB  > 10 mm in children born in countries with a high prevalence of TB | K = 0.533 | Not calculated |
| **Brock I, 2004** | Denmark | TB contact in one school (cross-sectional) | 125 | Mean 17 yrs | QFT | 68% | NR | 10 mm | K= 0.866 | Not calculated |
| **Bruzzese E , 2009** | Italy | Immunocompromised cases (cross-sectional) | 80 | Median 12.5 yrs ( 2-24 yrs) | QFT-G-IT  T-SPOT.TB | 0% | T-SPOT.TB: 20%  QT-G-IT: 13.51 % | 5 mm | T-SPOT.TB: K= - 0.028  QFT-G-IT :K= -0.016 | Not calculated |
| **Bergamini BM, 2009** | Italy | Recent immigration or household contacts, or suspected pulmonary or lymph-nodal TB (cross-sectional) | 496 | Median 11.1 yrs Range 0-19 yrs | QFT-G  QFT-G-IT  T-SPOT-TB. | 0% | QFT-G  QFT-G-IT:4.76%  T-SPOT-TB:1.95% | 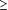5 mm suspected active TB or recent TB contacts; 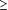10 mm in children <4 years of recent immigration | K= 0.43  QFT-G-IT: K= 0.35  T-SPOT.TB: K= 0.52 | Not calculated |
| **Carvahlo 2013** | Italy | Children contacts of a smear positive, drug-sensitive cavitary pulmonary TB in a hematology-oncology service (prospective study) | 18 | Median age 5,5 years (range: 1-18 years) |  | Not stated | QFT-G-IT: 17%; | Carvahlo 2013 | Italy | Children contacts of a smear positive, drug-sensitive cavitary pulmonary TB in a hematology-oncology service (prospective study) |
| **Chiappini E, 2012** | Italy | Children diagnosed with LTBI or acitve TB | 44 | Range:0-18 years | QFT-G-IT;  T-SPOT.TB | Not reported | Not reported | ≥ 5 mm in children in children in close contact with known or suspected contagious case of TB disease or for children suspected to have TB disease based on clinical evidence and/or chest radiograph; ≥ 10 mm for children born in countries with a high prevalence of TB and who recently immigrated | Not calculated | Not calculated |
| **Chiappini E, 2012** | Italy | Children with clinical suspicion of TB disease and/or in close contact with recently diagnosed cases of contagious TB disease and/or internationally adopted or recentky immigrated children coming from countries with a high prevalence of TB | 75 | Range:0-9 years | QFT-G-IT;  IFN-gamma and IL-2 ELISPOT | 17,33% | 1,33% | ≥ 5 mm in children in children in close contact with known or suspected contagious case of TB disease or for children suspected to have TB disease based on clinical evidence and/or chest radiograph; ≥ 10 mm for children born in countries with a high prevalence of TB and who recently immigrated | K=0.679 | Not calculated |
| **Chun JK, 2008** | Korea | Close TB contacts and casual TB contacts and control children (cross-sectional) | 227 | Range 0-15,8 yrs (median age 3,2 years) | QFT-G-IT | 100% | 7.5% | > 5mm | K= 0.19 (Close Contacts) K= 0.38 (Casual Contacts) | Not calculated |
| **Connell TG, 2006** | South Africa (Australian author) | Suspected active TB cases and latent TB cases (cross-sectional) | 106 | Median age in children with LTB: 9,2 yrs; active TB: 3.9 yrs | QFT-G | 51.49% | 17% | >15 mm in BCG vaccinated, >5 mm in known TB contacts, >10 mm for all others | K= 0.30 | Not calculated |
| **Connell TG,** **2008** | Australia | Suspected active TB and latent TB cases (cross –sectional) | 100 | Median 7 yrs. Range 1-19 | QFT-G-IT  T-SPOT.TB | 52.08% | QFT-G-IT:6%  T-SPOT.TB:20%^§^ | ;> 15 mm if BGC vaccinate; >10 mm in children with moderate risk factors; > 5 mm in high risk children | QFT-G- IT. K= 0.50  T-SPOT.TB k= 0.51 | K=0.83 |
| **Connell TG, 2010** | Australia | Children with suspected TB disease or latent TB infection (retrospective) | 875 | 9.1 years (range 25 days-18 years) | QFT-G (449)  QFT-G-IT (426) | NR | 13% | NR | Not calculated | Not calculated |
| **Connell TG, BMC Infect Dis. 2010;27:138** | South Africa | HIV- infected children with suspected TB disease (prospective) | 46 | Median age 22.6 months (IQR 12.4-59.6) | in house ELISPOT | 72% | definite TB group: 5%  probable TB group: 25% | > 5 mm | Not calculated | Not calculated |
| **Cruz AT, 2011** | USA | Children with or without risk factors for TB evaluated in 3 tuberculosis clinics (prospective study) | 210 | Range: 1 mo. – 18 years; median age: 8.6 years | T-SPOT.TB | 10.47% | 4.76% | ≥ 15 mm in children without risk factors;  ≥ 10 mm for children with chronic medical problems or exposure to people at high risk; ≥ 5 mm for children with suspected disease/immunocompromised or with identifiable source case | Not calculated | Not calculated |
| **Davies MA, 2009** | South Africa | Children with suspected TB (prospective) | 188 | Median 20 mo;Range 10-54.1 mo | In house ELISPOT (ESAT 6 CFP 10) | 76% | 9% | 5 mm in HIV infected children;10 mm in other children | Not calculated | Not calculated |
| **Dayal R, 2011** | India | Evaluation of 82 cases of active TB (68 pulmonary cases and 14 central-nervous.system cases); 48 disease matched controls and 20 TST+ controls (prospective) | 150 | Range: 0-18 years | QFT-G-IT | 33.0% in cases; 75.0% in controls | 11.8% in pulmonary cases and 50.0% in extrapulmonary cases | Not specified | Not calculated | Not calculated |
| **Debord C, 2011** | France | Retrospective analysis of 19 immunocompetent children with active TB | 19 | <6 years (median age: 1.52 years; range 0.29-5.36 years | QFT-G-IT | 84.2% | None | ≥5 mm in unvaccinated children;  ≥10 mm in BCG-vaccinated children | Not calculated | Not calculated |
| **Detjen AK, 2007** | Germany | Children with confirmed TB; NTM lymphadenitis; other respiratory tract infections (cross-sectional) | 73 | Median 39 mo Range 1-5 yrs | QFT-G-IT  T-SPOT.TB | 5.47% | QFT-G-IT: not reported  T-SPOT.TB:6.85% | > 5 mm | QFT –G-IT: K=0.48  T-SPOT.TB: K= 0.45 | K=0.91 |
| **Diel R, 2010** | Germany | close contacts of smear-positive index cases (prospective) | 141 | 10.4 ± 4.3 yrs | QFT-G-IT | 35.7% | 0% | > 5 mm. | Not calculated | Not calculated |
| **Dogra S, 2007** | India | Hospitalised cases with suspected TB disease or TB contacts (cross-sectional) | 105 | Median 6 years Range 1-12 years | QFT-G-IT | 92% | 0% | > 10 mm | QFT-G-IT : K=0.73 | Not calculated |
| **Dominguez J, 2008** | Spain | Active TB cases, or TB contacts or enrolled cases for screening of LTBI  (cross-sectional ) | 134 | Range 0-18 yrs | QFT-G-IT  T-SPOT.TB | 64.17% | T-SPOT.TB: 2.3 %  QFT-G-IT: 0.0% | > 5 mm | T-SPOT.TB: K= 0.34  BCG+:K= 0.12  BCG-: K=0.33  QFT -G–IT : K= 0.26  BCG+:K=0.08  BCG-: K=0.24 | Not calculated |
| **Ewer K,** **2003** | UK | School TB outbreak (cross-sectional) | 535 | Median 13.1 yrs Range 11-15 yrs | In house ELISPOT (ESAT-6 ; CFP-10) | 87.3% | NR | >15 mm in BCG vaccinated group;> 5 mm in unvaccinated BCG group | K = 0.72 | Not calculated |
| **Grare M, 2010** | France | Children at TB risk (recent contact or recent immigration) or with suspected active t TB isease (prospective) | 51 | Healthy contacts, mean age 5.8 years; LTBI children: mean age 5.6. Active TB children: mean age 6.5 years | QFT-G-IT | Healthy contacts: 39%. LTBI group: 62%. Active TB group: 43% | 33.3% | >10 mm for cases with risk factors for TB;other cases >15 mm | Not calculated | Not calculated |
| **Hansted E,** **2009** | Lithuania | Active TB, TB contacts and control children (cross sectional) | 120 | Median 14.0 yrs | T-SPOT.TB | 100% | NR | 10 mm | Not calculated | Not calculated |
| **Haustein T, 2009** | UK | Suspected active or latent TB cases  (high prevalence of immunodeficient patients) retrospective) | 269 | Median 7.3 yrs. (IQR 2.7-12.6) | QFT-G-IT | 51% | 35% | > 5 mm or >15 mm if BCG vaccinated | :K=0.71 | Not calculated |
| **HerrmannJL, 2009** | France | Latent B infection or active TB cases (prospective study) | 131 | Median 7.4 yrs  (2 mo-17 yrs.) | QFT-G-IT | 91% | NR | > 10 mm in non BCG  vaccinated or in BCG  vaccinated with  close TB contact  > 15 mm if BCG  vaccinated | K= 0.08 | Not calculated |
| **Hesseling AC, 2009** | South Africa | Recent household contacts with a TB case (cross sectional) | 29 | Mean 2.9 yrs; Range 0-5 yrs | QFT-G  T-SPOT.TB | 100% | QFT-G:14.3%  T-SPOT.TB:3.6% | > 10 mm | QFT-G :K = 0.78  T-SPOT .TB :K=-0.15 | K = -0.03 |
| **Hill PC,** **2006** | Gambia | TB household contacts (cross-sectional) | 287 | Median7.0 yrs. Range 0.5-14 yrs | In house ELISPOT (ESAT-6; CFP-10) | 43.6% | NR | > 10 mm | K= 0.62 | Not calculated |
| **Highuchi R, 2009** | Japan | Suspected TB cases  (prospective) | 61 | Range: 1 mo-16 yrs | QFT-G | 82.3% | NR | 10 mm | Not calculated | Not calculated |
| **Highuchi K, 2009** | Japan | Contact investigation in a primary school (prospective) | 308 | Range 8-12 yrs | GFT-G | 100%  close contacts  98.5% other contacts | 0.6% | 5 mm | Not calculated | Not calculated |
| **Kampmann B,** **2009** | UK | Active or latent TB cases (cross-sectional) | 209 | Mean 6.9 yrs; Range 0-16 yrs | QFT-G-IT  T-SPOT.TB | 68% | QFT-G-IT:6.80%  T-SPOT.TB: 8.74% | >15 mm | QFT-G-IT: K=0.57  T-SPOT.TB: K=0.46 | K= 0.66 (active TB)  K=0.15 (culture confirmed) |
| **Kasambira TS, 2011** | South Africa | Cross-sectional study with limited longitudinal follow-up in children household contacts of adults with newly diagnosed pulmonary TB | 270 | Range: 6 months-16 years | QFT-G-IT | 95% | 7% (19/270) | > 5 mm | K=0.58 (TST cutoff > 5 mm)  K= 0.54 (TST cutoff > 10 mm) | Not calculated |
| **Lewinsohn D, 2010** | Uganda | Household contacts (prospective) | 296 | Range 1-15 yrs | In house ELISPOT | 79% | < 5 years 7.2%  > 5 years 3.9% | > 5 mm. | K =0.40 (< 5 years)  K =0.39 (> 5 years) | Not calculated |
| **Liebeschuetz S, 2004** | South Africa | Suspected TB cases (prospective) | 293 | Median 50 months (IQR: 22-84 mo) | In house ELISPOT (ESAT-6 and CFP-10) | 95% | NR | > 0 mm in HIV-1 infected and > 15 mm in other children | K= 0.09 | Not calculated |
| **Lighter J, 2009** | USA | Children recruited from paediatric chest clinic, well-child clinic and paediatric inpatients ward (prospective) | 207 | Mean age 9 yrs Range 0-18 yrs | QFT-G-IT | 36% | 1.45% | 10 mm | K=0.19 | Not calculated |
| **Ling 2013** | South Africa | Hospitalized children with suspected pulmonary TB | 557 | Median age 22 months (IQR 12-53) | T-SPOT.TB | 85% | 5% | > 5 mm in HIV-infected children,  > 10 mm in other children | Not calculated | Not calculated |
| **Losi M, 2011** | Italy | Screening of immigrant children (perspective study) | 621 children screened with TST; in the 232 with positive or borderline result QFT-G-IT was perfomed | Range: 0-19 years | QFT-G-IT | Not reported; 100% in the four cases of active TB | 1.3% | 5-9 mm considered borderline; ≥10 mm considered positive | Not calculated | Not calculated |
| **Lucas M,** **2010 (65)** | Australia | Immigrated from Africa and Asia (Prospective study) | 524 | Median age in African children: 7.1 years (IQR: 3.6-11.0).  Median age in Asian children 7.8 years (IQR 2.8-11.9) | T-SPOT.TB  QFT-G-IT | 99.8% | T-SPOT.TB 2% (14% results were inconclusive, due to insufficient PBMCs or other technical problems)  QFT G-IT 15% | TST > 10 mm for all children originated from a high prevalence countries.TST > 15 mm for children aged less than 5 years and BCG-vaccinated. | T-SPOT.TB: K=0.45  QFT-G-IT: K=0.46 | K=0.78 |
| **Mandalakas AM,** **2008** | South Africa | HIV-infected children (cross-sectional) | 23 | Median 4.4 yrs | QFT-G    T-SPOT.TB | 91.3% | QFT-G:0 %  T-SPOT.TB:0% | >5 mm | T-SPOT.TB: K= -0.02;  QFT-G: K= 0.44 | K=:0.33 |
| **Mahomed H, 2011** | South Africa | Adolescents recruited from high schools in the study area (longitudinal study) | 5244 | Range: 12-18 years | QFT-G-IT | 93.8% | Not known because of exclusion | Agreement studied for different cut-offs: ≥ 5mm; ≥ 10 mm; ≥ 15mm | With TST cutoff ≥ 5mm: K= 0.70; with TST cutoff ≥ 10 mm: K=0.63; with TST cutoff ≥ 15mm: K=0.30 | Not calculated |
| **Mandalakas AM, 2012-2013** | South Africa | South-african children presenting fot out-patient care | 250 | Range: 3 months-15 years; median age: 39 months | QFT-G-IT; T-SPOT.TB | 93% | QFT-G-IT: 10 (4%);  T-SPOT.TB: 1 (0.4%) | ≥ 5mm in HIV-infected children; ≥ 10 mm in others | QFT-G-IT: K=0. 67 (95%CI 0.57;0.78)  T-SPOT.TB:  K= 0.53 (95%CI 0.41;0.65) | K= 0.75 (95%ci 0.65; 0.86 |
| **Markova R, 2011** | Bulgaria | Children hospitalized for clinical and/or radiological features compatible with active tuberculosis infection (prospective study) | 68 | Range: 0-16 years | QFT-G-IT | 100% | QFT-G-IT: 0% | ≥ 15mm | Not calculated | Not calculated |
| **Mendez-Echevarria A, 2011** | Spain | Immunocompetent children aged less than 15 years, who immigrated from TB endemic areas in the prior 2 years, children who were in contact with active TB, and children admitted with symptoms suggestive of TB (trasversal multicenter study) | 459 | Average age 4.73 +3.68 yrs | QFT-G-IT | 46,4% | QFT-G-IT: 8.8% | ≥10mm in immigrant children screened; ≥5mm in children in contact with active TB or with symptoms/radiological studies/pathological studies compatible with TB | QFT-G-IT: K= 0.91 in unvaccinated group; K=0.47 in BCG vaccinated group | Not calculated |
| **Molicotti P ,2008** | Italy | School TB outbreak (prospective) | 23 | Range 6-10 yrs | QFT-G-IT | 0% | 0 | 5 mm | Not calculated | Not calculated |
| **Moyo S, 2011** | South Africa | Children evaluated for TB disease in a high TB incidence setting | 400 children enrolled; 397 with both TST and QFT –G-IT results | < 3 years | QFT-G-IT | 100% | 5% | ≥10mm | K=0.79 | Not calculated |
| **Nakaoka H,** **2006** | Nigeria | Household contacts with smear positive and smear negative subjects ( cross-sectional) | 207 | Median 7.4 yrs Range 0-14 yrs | QFT-G-IT | 90% | 16% | 10 mm | K=0.246 (low risk)  K= 0.498 (high risk) | Not calculated |
| **Nenadic N, 2011** | Croatia | Children exposed to active TB and/or with positive TST | 59 | Range: 4-18 | QFT-G-IT | 100% | 0 | ≥10mm | Not calculated | Not calculated |
| **Nicol MP, 2005** | South Africa | Definite or probable TB cases (prospective) | 70 | Median 32 mo Range 3-154 mo | In house ELIPOT (ESAT-6; CFP-10) | NR | NR | 15 mm | Not calculated | Not calculated |
| **Nicol MP, 2009** | South Africa | exposure to tuberculosis or suspected active TB (cross-sectional) | 243 | Median 18 mo Range 0-NR | T-SPOT.TB | 100% | 6.17% | >10 mm | K= 0.548 | Not calculated |
| **Nkurunungi G, 2012** | Uganda | Children evaluated for LTBI | 907 | Aged 5 years | T-SPOT.TB | 100% | 5.4% | ≥10mm | K=0.28 at baseline, K=0.40 at follow-up | - |
| **Noorbakhsh S, 2011** | Iran | Young household contacts of immunocompetent cases of proven pulmonary tuberculosis | 59 | Aged less than 20 years | QFT-G | 100% | Not reported | ≥10mm | K=0.43 | Not calculated |
| **Nsutebu E, 2008** | UK | Screening in students (prospective) | 190 | Range: 13-14 yrs | QFT-G-IT | NR | NR | NR | Not calculated | Not calculated |
| **Ohno H, 2008** | Japan | Hospitalized cases with TB contact (nurse) and their visitors (prospective) | 28 | Children under 10 years: mean 2.5 yrs | QFT-G | 11.90% | 2.75% | > 5 mm in BCG unvaccinated s;>15 mm in BCG vaccinated | Not calculated | Not calculated |
| **Okada K, 2008** | Cambodia | Household contacts of TB cases (cross-sectional) | 195 | Range: 0-5 yrs | QFT-G-IT | 88% | 4.14% | > 10 mm | K= 0.626 | Not calculated |
| **Pavic C, 2011** | Croatia | BCG-vaccinated chidren with History of exposure to active TB (prospective) | 142 | Range: 0-5 years; average age 29 + 16 months | QFT-G-IT | 100% | 0.7% | > 10 mm | K= 0.591 | Not calculated |
| **Petrucci R, 2008** | Nepal and Brazil | TB case contacts (cross-sectional) | 259 | Median 8.5 yrs (2 mo- 15 yrs) | QFT-G-IT | 96.5% | 3.42% | > 10 mm | K= 0.73 (Brazil)  K= 0.80 (Nepal) | Not calculated |
| **Riazi S, 2012** | USA | Children evaluated because of an ambiguous diagnostically interpretable TST, referred mainly by pediatricians from private and public health clinics (prospective) | 517 | Range: 1 month-18 years | QFT-G | 68.7% | 7.15% | > 10 mm | Not calculated | Not calculated |
| **Richeldi L, 2004** | Italy | Newborns, TB hospital contacts (prospective) | 41 | Range: 0-4 days | In house ELISPOT (ESAT-6 and CFP-10) | 9.8% | NR | > 5 mm | Not calculated | Not calculated |
| **Rose MV, 2012** | Tanzania | Children with signs and symptoms suspect for TB (prospective study) | 211 | Range: 0-15 years; mean age 4.4 ± 3.8 | QFT-G-IT | 93% | 27% | > 5 mm in HIV-positive children; > 10 mm in others | Not calculated | Not calculated |
| **Rutherford ME, 2012** | Indonesia | Evaluation of children exposed to sputum smear and chest-X-ray positive adult TB patients at household and neighbourhood level, recrited in a community-based lung clinic (prospective) | 299 household-exposed and 72 neighborhood exposed children | Range: 6 months-9 years; Median age: 61 months | QFT-G-IT | 73% | 3% | > 10 mm | K=0.58  (K=0.59 in household contacts; K=0.40 in neighbourhood contacts) | Not calculated |
| **Shah M, 2011** | South Africa | Prospective study with 6 months longitudinal follow-up of children household contacts of adults with newly diagnosed pulmonary TB previously evaluated | 196 | Range: 6 months-16 years; median age 6 years (IQR:3-9years) | QFT-G-IT | 95% | 7.7% | > 5 mm | K range= 0.11-0.33 | Not calculated |
| **Soborg B, 2010** | Greenland, Alaska, Canada | Survey in school-aged children (prospective) including 4 cases if active tuberculosis | 2218 | 11.4 ± 3.1 yrs. | QFT-G-IT | 34.8% | 1.0% | >12 mm | K=0.79 (all children)  K=0.68 (BCG vaccinated)  K=0.74 (Not vaccinated) | Not calculated |
| **Soysal A, 2005** | Turkey | Household Tb contacts (cross sectional) | 979 | Median :7 yrs (IQR 0-16) | In house ELISPOT (ESAT-6 and CFP-10) | 79% | NR | >10 mm | K= 0.76 (no BCG scar),  K=0.50 (BCG scar) | Not calculated |
| **Soysal A, 2008** | Turkey | Healthy children aged between 6 and 10 years recruited during school usual TST screening before the second dose of BCG | 209 | Mean age 8.4 (SD 0.68 years | T-SPOT.TB | 90% | Not reported | >15 mm | Not calculated | Not calculated |
| **Stavri HR, 2010** | Romania | Hospitalized children with TB | 60 | Range 1-18 years; median age: 9.44 ± 5.27 | QFT-G-IT | 100% | Yes | >10 mm | K range: 0.03-0.05 | Not calculated |
| **Stefan DC, 2010** | South Africa | Children newly diagnosed with cancer, in area at high risk for TB disease | 34 | Median: 7 years (range 2 mo.-15 yr.s) | T-SPOT.TB  QFT-G-IT | 99% | QFT-G-IT:14%  T-SPOT.TB: 18.5% | >10 mm | T-SPOT.TB: K= 0.33  QFT-G-IT: K=0.26 | K=0.26 |
| **Sun L, 2010** | China | Children with active TB and non-tubercolous children (cross-sectional) | 125 | Mean age 7,1 years (SD 4,5) | TSPOT | 77.6% | Not reported | >10 mm | K=0.203 active TB patients | - |
| **Tavast E, 2009** | Finland | Children at risk for TB infection (retrospective) | 27 | Median 9 yrs Range 0-18 yrs | QFT-G-IT  T-SPOT.TB | 68.69% | 0% | > 10 mm in BCG-vaccinated children;> 5 mm in not-vaccinated children | QFT-G-IT: K= 0.76  T-SPOT.TB: K=0.86 | K=0.89 |
| **Tsolia M,** **2010** | Greece | Children at risk for TB infection (cross sectional) | 286 | aged <15 years | QFT-G-IT | 49.4% | 5.94% | > 10 mm for BCG vaccinated children and unvaccinated children with no risk factors; > 5 for unvaccinated BCG children with active TB or TB contacts | K=0.46 | Not calculated |
| **Tsiouris SJ, 2006** | South Africa | Schoolchildren at high risk for LTB (cross-sectional) | 184 | Median 9 yrs Range 5-15 yrs | QFT-G-IT | 72.3% | 0% | >10 mm | K=0.56 | Not calculated |
| **Taylor REB, 2008** | UK | Children at risk for TB infection (cross sectional) | 130 | Median: 10 yrs. Range :4 mo-16 yrs | QFT-G-IT | 47% | 6% | > 15 mm in BCG-vaccinated children; > 5 mm in not-vaccinated children | Not calculated | Not calculated |
| **Thomas TA, 2010** | Bangladesh | Cross sectional study | 251 | 13.1 years + 1.25 years | QFT-G-IT | 79.1% | 24.5% | >10 mm | K=0.55 | Not calculated |
| **Thomas B, 2011** | UK | Asymptomatic children contacts of active TB screened for diagnosis of LTBI (retrospective study) | 283 | Range: 0-16 years; mean age 5.3 (SD 4.1) years | QFT-G-IT | 71.7% | 6.4% | ≥6 mm for unvaccinated; ≥15 mm for BCG vaccinated children | K= 0.70 | Not calculated |
| **Uluk 2013** | Papua Guinea | Children with suspected TB (prospective, cross-sectional study) | 216 | Range: 1 month-12 years | QFT-G-IT | 75% | 13% | > 10 mm in BCG-vaccinated children; > 5 mm in not-vaccinated children | K=0,70 | Not calculated |
| **Warier A,** **2009** | India | Children with TB infection on anti-tubercular therapy + healthy controls (cross-sectional) | 143 | Range: 0-18 yrs | T-SPOT.TB | 25.58% | 16.1% | > 10 mm | Not calculated | Not calculated |
| **Winje AB, 2008** | Norway | School-screening (Cross sectional) | 519 | 15 yrs | QFT-G-IT | 46.2% | NR | >6 mm | Not calculated | Not calculated |
| **Yassin MA, 2011** | Ethiopia | Evaluation of children with symtoms of TB, children in contact with adults with pulmonary TB and community controls(C ross-sectional study) | 322 children with symtoms of TB, 335 children in contact with adults with pulmonary TB and 156 community controls | Range: 1-15 years; median age: 5-8 years | QFT-G-IT | 67,9 | 22.01% | ≥10 mm | K=0.49 in confirmed TB;  K=0.40 in probable TB;  K=0.23 in unlikely TB;  K=0.39 in contacts  K=0.24 in controls | Not calculated |

Legend: K; kappa statistic; TST: Tuberculin Skin test; IGRA: interferon-γ release assays; QFT-G-IT: QuantiFERON-TB Gold In-Tube; ELISPOT: ex vivo enzyme-linked immunospot assay for gamma-interferon, LTBI: latent tuberculosis infection; BCG: Bacille Calmette-Guérin
